# Maternal regulation of the vertebrate oocyte-to-embryo transition

**DOI:** 10.1371/journal.pgen.1011343

**Published:** 2024-07-25

**Authors:** Ricardo Fuentes, Florence L. Marlow, Elliott W. Abrams, Hong Zhang, Manami Kobayashi, Tripti Gupta, Lee D. Kapp, Zachary DiNardo, Ronald Heller, Ruth Cisternas, Priscila García-Castro, Fabián Segovia-Miranda, Felipe Montecinos-Franjola, William Vought, Charles E. Vejnar, Antonio J. Giraldez, Mary C. Mullins

**Affiliations:** 1 Department of Cell and Developmental Biology, University of Pennsylvania, Perelman School of Medicine, Philadelphia, Pennsylvania, United States of America; 2 Departamento de Biología Celular, Facultad de Ciencias Biológicas, Universidad de Concepción, Concepción, Chile; 3 Department of Cell, Developmental and Regenerative Biology, Icahn School of Medicine Mount Sinai, New York, New York, United States of America; 4 Department of Biology, Purchase College, State University of New York, Purchase, New York, United States of America; 5 Division of Developmental Biology, Eunice Kennedy Shriver National Institute of Child Health and Human Development, National Institutes of Health, Bethesda, Maryland, United States of America; 6 Laboratory of Cell Structure and Dynamics, National Institute on Deafness and Other Communication Disorders (NIDCD), National Institutes of Health, Bethesda, Maryland, United States of America; 7 Department of Genetics, Yale University School of Medicine, New Haven, Connecticut, United States of America; HudsonAlpha Institute for Biotechnology, UNITED STATES OF AMERICA

## Abstract

Maternally-loaded factors in the egg accumulate during oogenesis and are essential for the acquisition of oocyte and egg developmental competence to ensure the production of viable embryos. However, their molecular nature and functional importance remain poorly understood. Here, we present a collection of 9 recessive maternal-effect mutants identified in a zebrafish forward genetic screen that reveal unique molecular insights into the mechanisms controlling the vertebrate oocyte-to-embryo transition. Four genes, *over easy*, *p33bjta*, *poached* and *black caviar*, were found to control initial steps in yolk globule sizing and protein cleavage during oocyte maturation that act independently of nuclear maturation. The *krang*, *kazukuram*, *p28tabj*, and *spotty* genes play distinct roles in egg activation, including cortical granule biology, cytoplasmic segregation, the regulation of microtubule organizing center assembly and microtubule nucleation, and establishing the basic body plan. Furthermore, we cloned two of the mutant genes, identifying the *over easy gene* as a subunit of the Adaptor Protein complex 5, Ap5m1, which implicates it in regulating intracellular trafficking and yolk vesicle formation. The novel maternal protein Krang/Kiaa0513, highly conserved in metazoans, was discovered and linked to the function of cortical granules during egg activation. These mutant genes represent novel genetic entry points to decipher the molecular mechanisms functioning in the oocyte-to-embryo transition, fertility, and human disease. Additionally, our genetic adult screen not only contributes to the existing knowledge in the field but also sets the basis for future investigations. Thus, the identified maternal genes represent key players in the coordination and execution of events prior to fertilization.

## Author summary

The oocyte-to-embryo transition consists of the coordinated regulation of multiple molecular processes acting in the late oocyte, egg and early zygote. This transcriptionally silent period requires the precisely timed function of maternally-supplied gene products during oogenesis. However, knowledge of their molecular nature and *in vivo* function remains incomplete. Importantly, defects associated with the oocyte-to-embryo transition in humans cause reduced female reproductive success, in addition to a possible increased incidence of miscarriage. The mutants reported here provide access to maternal factors regulating the processes that prepare an oocyte for reproductive competence and embryogenesis. We have identified essential regulators of yolk granule formation, cortical granule biology, and microtubule organizing assembly control. Specifically, we found that the highly conserved maternal Ap5m1 protein regulates yolk granule maturation, which generate essential nutrients and immunity for growth and development in oviparous animals. We characterized the Krang factor, which regulates aspects of egg activation likely by modulating the secretory pathway. The mutants presented here represent attractive genetic models to investigate the molecular and cell biological mechanisms that control the oocyte-to-embryo transition, as well as reveal a collection of genetic factors indispensable for reproduction and survival. Importantly, knowledge of their genetic underpinnings and biological importance in reproduction will also pave the way to investigate the evolution of maternal genes during vertebrate development.

## Introduction

Animal embryonic development depends on factors endowed to the oocyte during oogenesis. These molecules make it competent to mature into an egg that will activate and subsequently undergo embryonic development by a process called the oocyte-to-embryo transition. This gradual and tightly regulated developmental program requires the function of gene products, called maternal factors, that are loaded into the egg during oogenesis (reviewed in [1–5]). Oocyte maturation and egg activation are fundamental to the oocyte-to-embryo transition, but how these events are molecularly regulated is not well understood. In fact, the molecular identity and function of most of the maternal factors orchestrating the embryonic program in vertebrates remain unknown, highlighting the need for developing methods to identify and study them.

In many oviparous organisms, including zebrafish, the oocyte accumulates maternal Vitellogenin (Vtg) protein via receptor-mediated endocytosis [6]. Maternal Vtg, a conserved phospholipoglycoprotein, is the primary yolk protein and a vital nutrient for embryonic development in many egg-laying species, including platypus, but not placental mammals [7]. Once Vtg is internalized, it is partially processed in endosomes and then stored as yolk globules (YGs) during oogenesis (reviewed in [8]). YGs are modified lysosomes and contain hydrolases that process the yolk proteins; however, unlike typical lysosomes, YGs do not immediately process their contents [8,9]. As important lysosomal vesicles, YGs and the mechanism behind their formation and function could have clinical implications, since the etiology of most vesicle trafficking- and lysosomal-related defects in human disease remain unresolved (reviewed in [10–12]).

During late stages of oogenesis, nuclear maturation and cytoplasmic changes (cytoplasmic maturation) together produce a viable egg, including a shift in the metabolic and optical properties of YGs [13,14]. During oocyte maturation in vertebrates, the oocyte arrests in meiosis II until ovulation of the egg and its subsequent activation (reviewed in [15–18]). Egg activation is typically initiated by fertilization; while in zebrafish contact of the egg with a hypotonic solution induces activation. Following the activation stimulus, a wave of calcium release initiated at the sperm entry point triggers the exocytosis of secretory vesicles called cortical granules (CGs). Additionally, meiosis II resumes and is completed with extrusion of the second polar body [19–21]. In zebrafish, the calcium wave also triggers the cytoplasm to segregate to the animal pole to form the single cell blastodisc.

In metazoans, CGs are synthesized, transported and docked at the oocyte cortex during oogenesis (reviewed in [22]). The CGs contain enzymes, glycosylated components and structural proteins that remodel the vitelline envelope surface, driving elevation and hardening of the vitelline envelope (referred to as the chorion in zebrafish and zona pellucida in mammals) during egg activation (reviewed in [22]). CG exocytosis prevents polyspermy and provides protection to the prospective embryo in organisms of different taxa (reviewed in [22–24]). Abnormalities in CG exocytosis and the vitelline envelope can cause a failure in fertilization, early embryonic development, and/or implantation [25–27]. Therefore, advances in understanding the molecular mechanisms governing CG exocytosis and vitelline envelope formation are also relevant to human assisted reproductive technology (ART) [28].

In animal systems, cells divide in the absence of cell growth during early embryogenesis. Thus, the spatial and temporal reorganization of maternal factors into specific cytoplasmic domains within the egg and zygote prior to the first cell division, is critical for coordinating the many distinct activities carried out by the early embryo [29–31]. In zebrafish zygotes, spatial reorganization and animal-directed flow of cytoplasm during cytoplasmic segregation form a prominent cytoplasmic domain and the site of the future embryonic cleavages, the blastodisc [19,29,32,33].

Within the blastodisc, the zygotic nucleus, centrosome and mitotic spindle form as the first cell cycle initiates [29,34,35], in the transition from meiosis to mitotic cleavage divisions. The maternal factors guiding the spatiotemporal transport of morphogenetic determinants across the egg and zygote, and its coordination with critical events in the shift from meiosis to mitosis remain largely unknown. The zebrafish egg and zygote constitute an excellent model system to study this transition since they are easily manipulated due to their large size and external development. These features make the zebrafish suitable for the study of highly dynamic events during the oocyte-to-embryo transition, such as the establishment of the first cell cycle.

Here we report a forward genetic adult screen to identify novel maternal genes functioning in the oocyte-to-embryo transition in the zebrafish. We identified 9 maternal-effect mutants falling into two classes of phenotype, those with defects in oocyte maturation, and those with defects in egg activation and subsequent embryogenesis. Each of these genes is required for aspects of YG formation and maturation, CG biology, cytoplasmic segregation, or the regulation of MTOC assembly and nucleating activity. This collection of vertebrate mutants represents a valuable resource for understanding the molecular regulation of the oocyte-to-embryo transition, providing genetic entry points and models of infertility or poor reproductive success.

## Results

### Identification of zebrafish oocyte-to-embryo transition mutants

To isolate mutations in genes regulating the oocyte-to-embryo transition in zebrafish, we induced mutations with the chemical mutagen N-ethyl-N-nitrosourea (ENU) and conducted a four-generation adult maternal-effect screen for egg and embryo phenotypes [13,36]. We examined 716 mutagenized genomes, and identified 9 recessive oocyte-to-embryo transition maternal-effect mutants (Table 1), which failed to produce eggs that were competent to undergo early embryonic development. In addition, we identified 9 maternal-effect mutants disrupting the cleavage stage of embryogenesis in zebrafish [37]. Five of the oocyte-to-embryo transition mutants displayed phenotypes indicating a primary defect during oogenesis, including one new mutant allele of the *over easy* (*ovy*^*p37ad*^) gene [13]. Four additional mutant genes were identified that disrupt egg activation.

**Table 1:** Zebrafish Maternal-effect Oocyte-to-Egg Transition and Egg Activation Mutant Genes.

Mutant Class	Gene/allele	Molecular identity^a^	Chromosomal Locus^b^
Oocyte to egg transition	*p33bjta*	ND	ND
	*over easy* ^*p35aluc*^	*ap5m1*	Chr 17, z8980
	*over easy* ^*p37ad*^	*ap5m1*	Chr 17, z8980
	*poached* ^*p26ahubb*^	ND	Chr 17, z21194
	*black caviar* ^*p25bdth*^	ND	Chr 5, z3804
	*black caviar* ^*p26ahubg*^	ND	Chr 5, z3804
Egg activation	*krang* ^*p30ahub*^	*kiaa0513*	Chr 18, z24123
	*kazukuram* ^*p26thbd*^	ND	Chr 8, z8703
	*spotty* ^*p08bdth*^	ND	Chr 2, z24206
	*p28tabj*	ND	ND

ND = Not yet determined

(a) *ap5m1* = adaptor related protein complex 5 subunit mu 1; *kiaa0513* = uncharacterized gene

(b) Chromosome (Chr), closely linked marker

### Oocyte maturation genes

**Fig 1:**
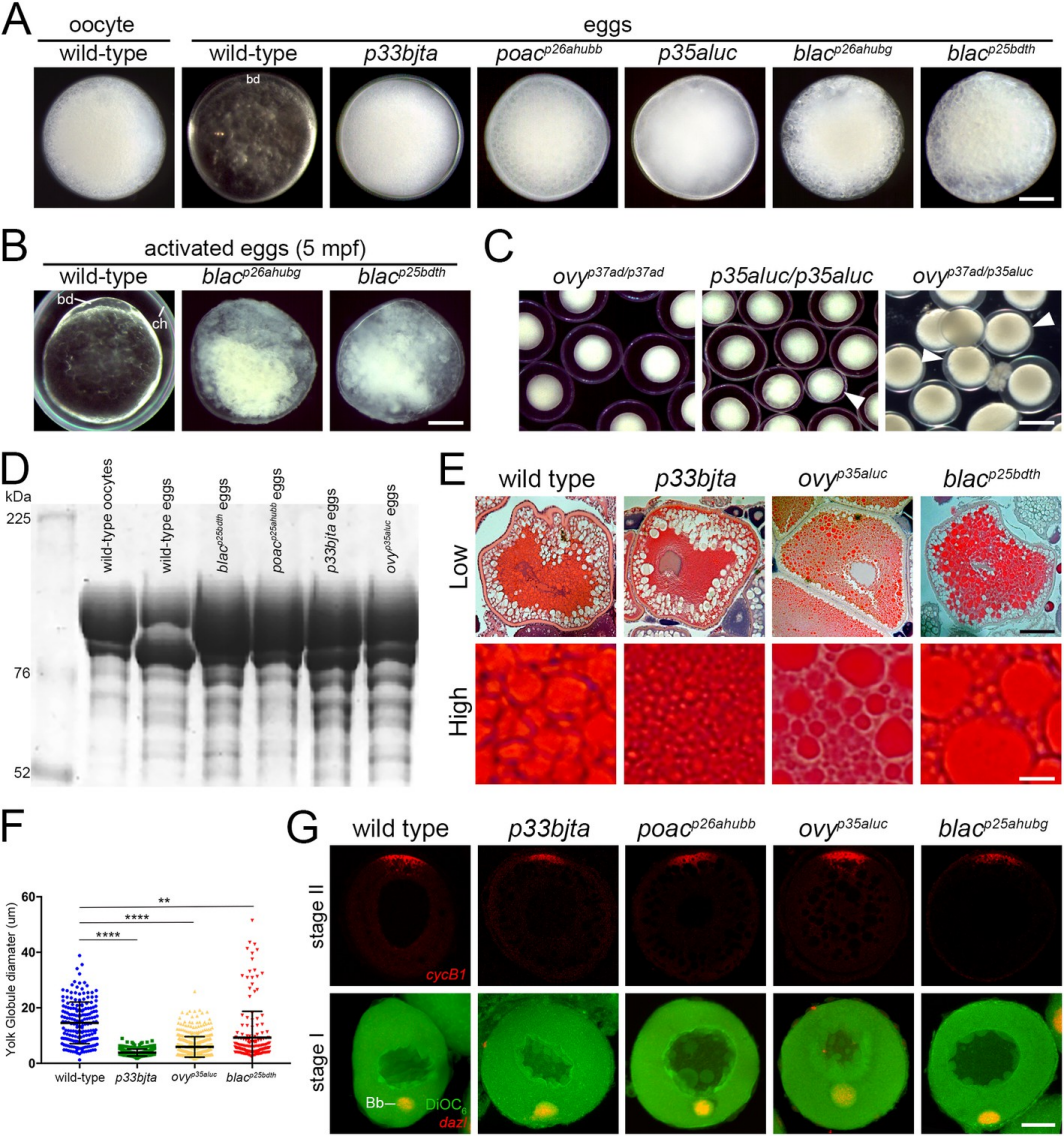
Zebrafish oocyte maturation mutants. **A.** Wild-type stage IV oocyte and egg in incident light. Eggs of *p33bjta* (1570 from 21 females), *poac*^*p26ahubb*^ (1620 from 35 females), and *p35aluc* (2088 from 25 females) resemble wild-type oocytes in their opacity, while eggs of *p26ahubg* (546 from 8 females) and *blac*^*p25bdth*^ (1020 from 17 females) were degenerated. **B.** Images of 5 minutes post-egg activation (mpa) wild-type (n = 68), *p26ahubg* (n = 56) and *blac*^*p25bdth*^ (n = 61) eggs, showing lysed mutant eggs. bd, blastodisc. **C.** Non-complementation test showed that females carrying the *ovy*^*p37ad*^ mutation in trans to the *p35aluc* mutation produced opaque mutant eggs (n = 1382 from 9 females), showing that *p35aluc* is a new *ovy* allele. **D.** Coomassie stained gel of major yolk proteins in wild-type and mutant eggs showing that the cleavage of yolk protein is impaired. kD, kilodaltons. **E.** Low (top row) and high (bottom row) magnification histological sections of wild-type and mutant stage III oocytes stained with H&E revealing YG sizes. Scale bar = 96 μm (top row) and 16 μm (bottom row). **F.** Scatter plots of YG diameters measured in a defined region of the oocyte. The mean diameter of the YGs in the mutants was significantly different to that of wild-type oocytes (WT = 14.6 μm, *p33bjta* = 3.87 μm, *p35aluc* = 5.95 μm, *p25bdth* = 9.25 μm). The number of YGs analyzed in this study was: wild type (n = 188 from 4 oocytes), *p33bjta* (n = 320 from 2 oocytes), *ovy*^*p35aluc*^ (n = 503 from 2 oocytes), and *blac*^*p25bdth*^ (n = 177 from 2 oocytes). Data are means ± SEM. ****p<0.0001 and **p = 0.0066 in parametric statistical Tukey’s test. ns, not significant. **G.** Confocal z-projections of isolated acid fixed oocytes analyzed by fluorescent *in situ* hybridization and also DiOC_6_ staining (green) in lower panels. In stage II oocytes, *cycB1* transcript localized to the animal pole in wild-type (n = 32), *p33bjta* (n = 22), *poac*^*p26ahub*^ (n = 47), *ovy*^*p35aluc*^ (n = 38), and *blac*^*p25bdth*^ (n = 28) oocytes. In stage I oocytes, *dazl* mRNA co-localized with DiOC_6_ to the vegetal Balbiani body (Bb) in wild-type (n = 32), *p33bjta* (n = 23), *poac*^*p26ahub*^ (n = 63), *ovy*^*p35aluc*^ (n = 31), and *blac*^*p25bdth*^ (n = 29) oocytes. Scale bar = 35 μm.

The largest group of mutants identified in our screen exhibits defects in oocyte maturation (Fig 1A). In zebrafish, the clarity of the yolk is a cytoplasmic indicator of this process. Prior to oocyte maturation in wild type, the yolk is opaque and the bulk of the cytoplasm is intermingled between the YGs in the oocyte. During oocyte maturation, the yolk proteins are cleaved, generating the translucency of the egg (Fig 1A) [38]. Three mutants reported here, *p33bjta*, *p26ahubb*, and *p35aluc*, produced eggs that were opaque and lacked a blastodisc, more closely resembling wild-type oocytes than eggs (Fig 1A). The other two mutants of this class, *p26ahubg* and *p25bdth*, only produced degenerated eggs (Figs 1B and S1A), indicating a more severe or distinct defect in the oocyte-to-egg transition.

To determine if the mutations represent alleles of the same or distinct genes, we determined the chromosomal positions of each mutation (Table 1). The *p35aluc and p26ahubb* mutations mapped to a similar chromosomal location as the *ovy*^*p37ad*^ mutation, which also causes an opaque egg phenotype [13]. Hence, we conducted complementation tests between these alleles. We found that *p35aluc* failed to complement *ovy*^*p37ad*^ (Fig 1C), indicating that *p35aluc* is an allele of *ovy*^*p37ad*^. In contrast, we found that *p26ahubb* complemented *ovy*^*p37ad*^, indicating that this mutation disrupts a different gene, which we named *poached* (after a type of cooked egg_;_ hereafter called *poac*^*p26ahubb*^). Among the mutants identified in this screen, only *p35aluc* (hereafter called *ovy*^*p35aluc*^) represented a new allele of a previously reported zebrafish maternal-effect gene. We did not find linkage between the *p33bjta* mutation and the SSLP markers tested. However, our analysis indicates that the *p33bjta* mutation is not linked to the chromosomal intervals of the other opaque egg mutations reported here or previously [13], suggesting that it disrupts a distinct gene.

Homozygous females of the *p25bdth* and *p26ahubg* mutations produced an identical phenotype, lysed eggs at egg laying (Figs 1B and S1A). We found that both mutations mapped to the same chromosomal position (Table 1) and failed to complement each other (see Materials and Methods), thus indicating that these two alleles disrupt the same gene (hereinafter called *black caviar* (*blac*)).

### Oocyte cytoplasmic maturation of YGs

The zebrafish stage III oocyte is opaque due to the accumulation of membrane-enclosed crystalline yolk; however, at the end of oocyte maturation the yolk becomes non-crystalline and translucent [16,38]. This process is driven by the proteolytic processing of the major yolk proteins during oocyte maturation in stage IV oocytes, changing their refractive index from opaque to translucent. The persistent opaque yolk in these mutant eggs suggests that cleavage of the major yolk proteins during oocyte maturation is impaired. To investigate this directly, we prepared cytoplasmic extracts from wild-type immature stage IV vitellogenic oocytes and eggs, and compared the yolk protein profiles to those of the oocyte maturation class of mutant eggs. We found that the yolk protein composition of mutant eggs of *blac*^*p25bdth*^, *poac*^*p26ahubb*^, *p33bjta* and *ovy*^*p35aluc*^ resembled wild-type oocytes rather than wild-type eggs (Fig 1D), indicating that cleavage of the vitellogenic proteins was indeed defective during oocyte maturation in these mutants.

To further understand the YG maturation defect, we asked whether YG morphology and integrity was also affected. We therefore examined the size of YGs in wild-type and mutant stage III oocytes in hematoxylin and eosin (H&E) stained histological sections (Fig 1E). We found that the YGs of wild-type stage III oocytes displayed a mean diameter of 14.6 μm (Fig 1F). In *p33bjta*, *ovy*^*p35aluc*^ and *blac*^*p25bdth*^ mutants, the YGs were on average 3.7-, 2.5-, and 1.6-fold smaller than in wild type, respectively (Fig 1E and 1F). The *p33bjta* phenotype was more severe than *ovy*^*p35aluc*^, displaying a much greater amount of small YGs and no large YGs (Fig 1E and 1F). In *blac*^*p25bdth*^ mutant oocytes numerous, smaller YGs were present, but some large YGs also formed, with a few larger than in wild type (over 40 μm) (Fig 1E and 1F). Thus, quantification of YG size confirmed a significant difference between wild type and mutants (Fig 1F), likely associated with altered YG formation during oogenesis in *ovy*^*p35aluc*^ and *blac*^*p25dbth*^ mutants.

Histological sections of earlier stage I and II oocytes revealed no overt differences between mutants and wild type (S1B Fig). H&E staining indicated that previtellogenic oocytes were normal; cortical granules (CGs) accumulated and later localized to the oocyte cortex in *ovy*^*p35aluc*^, *p33bjta*, and *blac*^*p25bdth*^ mutant stage III oocytes (S1B Fig).

These data indicate that YG size was severely compromised in *ovy*^*p35aluc*^ and *p33bjta* mutants; thus, suggesting that the products of the *ovy* and *p33bjta* genes promote the formation of large YGs. Taken together, the anomalies observed in these mutants strongly suggest that *p33bjta*, *ovy* and *blac* regulate the initial steps of YG formation and sizing, and yolk protein processing, independent of other aspects of oocyte cytoplasmic maturation.

### Oocyte maturation mutants exhibit defects in egg activation and early embryogenesis

All mutants of this class produced eggs that failed to segregate cytoplasm to the animal pole to form the blastodisc and showed no overt signs of egg polarity (Fig 1A–1C). Therefore, we examined markers of the animal-vegetal axis to investigate whether failed blastodisc formation was due to a defect in oocyte polarity. We found that, like in wild type, c*yclinB1* (*cycB1*) mRNA localized to the animal pole of *p33bjta*, *ovy*^*p35aluc*^, *poac*^*p26ahubb*^ and *blac*^*p25bdth*^ stage II oocytes and *deleted in azoospermia-like* (*dazl*) mRNA localized to the vegetal Balbiani body of stage I oocytes (Fig 1G). Therefore, impaired oocyte polarity does not cause the lack of animal-ward segregation of cytoplasm that forms the blastodisc.

**Fig 2:**
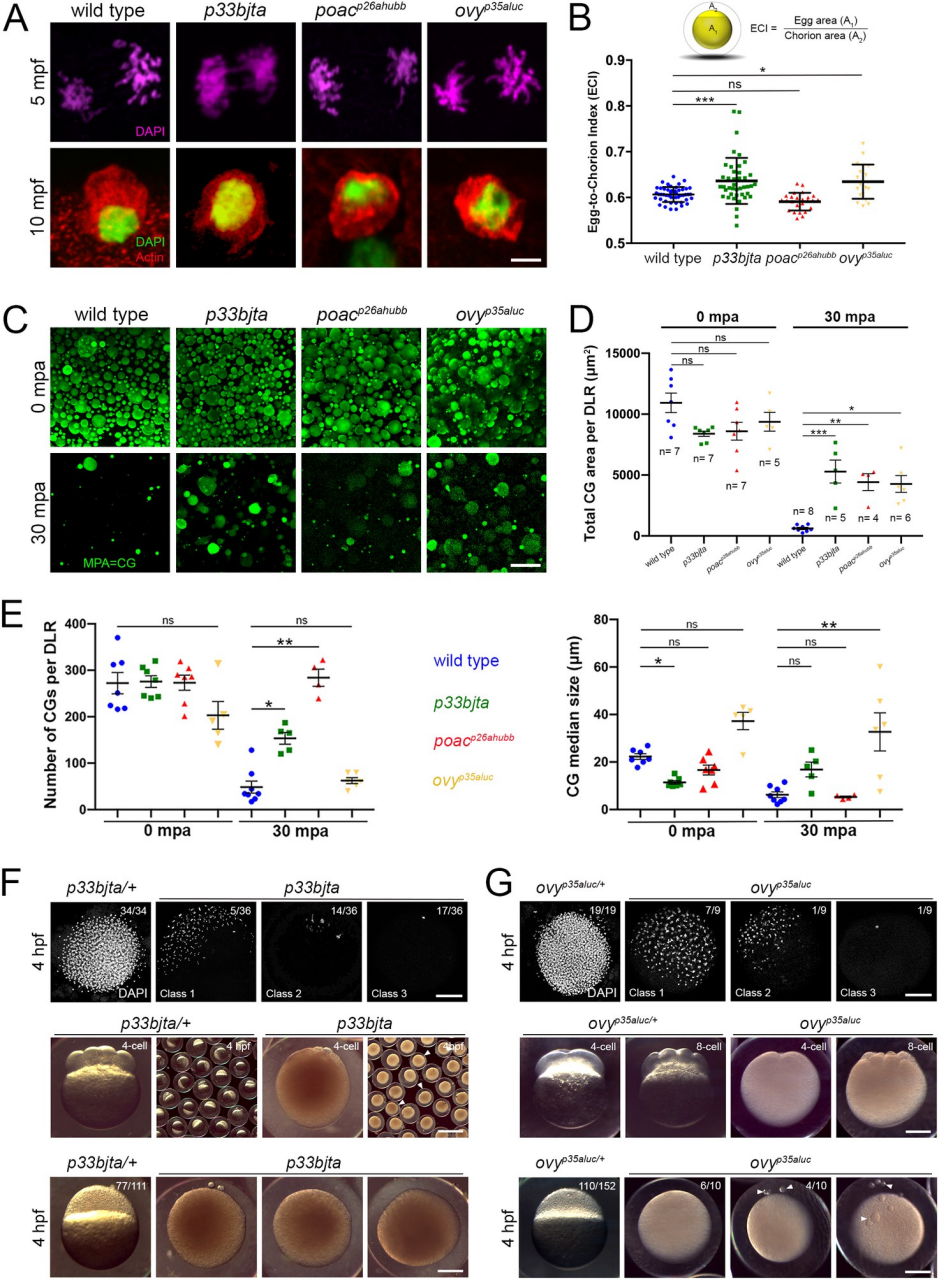
Meiosis II completion and egg activation defect in dark eggs mutants. **A.** Confocal micrographs of the animal pole show anaphase of meiosis II at 5 mpa and the extruded polar body at 10 mpa. Top row: DAPI-stained wild-type (n = 24), *p33bjta* (n = 14), *poac*^*p26ahubb*^ (n = 22) and *ovy*^*p35aluc*^ (n = 21) sister chromatids during meiotic anaphase II. Bottom row: Phalloidin-stained actin (red) and DAPI staining (green) of second polar bodies in wild-type (n = 14), *p33bjta* (n = 9), *poac*^*p26ahubb*^ (n = 12) and *ovy*^*p35aluc*^ (n = 10) activated eggs. Scale bar = 4 μm. **B.** Egg-to-chorion index (ECI) is the ratio of egg area (A_1_) to chorion area (A_2_) as a measure of chorion elevation. Scatter plots of the ECI measured in activated eggs at 30 mpa. The wild-type egg ECI value (ECI = 0.61; SD = 0.02; n = 65) defines the numerical value for normal chorion elevation. The ECI for the eggs from *p33bjta* (ECI = 0.64; SD = 0.05; n = 44, 2 females) and *ovy*^*p35aluc*^ (ECI = 0.63; SD = 0.03; n = 20, 2 females) mutant females are significantly different to wild type, while the eggs of *poac*^*p26ahubb*^ (ECI = 0.59; SD = 0.02; n = 25, 2 females) mutant females show a mean value similar to wild type. SD = standard deviation. Data are means ± SD. *p = 0.0131 and ***p = 0.0004 in parametric statistical Tukey’s test. **C.** Confocal z-projections (5 μm depth) of acid fixed and MPA stained wild-type and mutant activated eggs. In wild-type (n = 60), *p33bjta* (n = 55), *ovy*^*p35aluc*^ (n = 25), and *poac*^*p26ahubb*^ (n = 27) eggs, numerous large and small CGs were distributed throughout the cortex at 0 mpa. At 30 mpa, wild-type (n = 21), *p33bjta* (n = 28), *poac*^*p26ahubb*^ (n = 20) and *ovy*^*p35aluc*^ (n = 18), eggs displayed fewer CGs, indicating that they were released following activation. However, some CGs persisted in *p33bjta* (5/28), *poac* (11/20) and *ovy*^*p35aluc*^ (6/18) mutant eggs. **D.** Scatter plots of CG retention measured in a determined lateral region (DLR) of activated wild-type and opaque mutant eggs. The total area of retained CGs per total DLR area in all dark egg mutants was significantly increased compared to wild type at 30 mpa. n, number of eggs used in this analysis from 3 females. Data are means ± SEM. *p = 0.0249, **p = 0.0485 between wild type and *poac*, and ***p = 0.0042 between wild type and *p33bjta* in a nonparametric statistical Kruskal-Wallis test. ns, not significant; mpa, minutes post-activation. **E.** Scatter plots of CG number (top graph) and median size (bottom graph) measured in the DLR of the wild-type and dark activated eggs. The average number of CGs in the mutants was comparable to wild type at 0 mpa but significantly different in the *p33bjta* and *poac*^*p26ahubb*^ mutants at 30 mpa. Unactivated (wild type (n = 7), *p33bjta* (n = 7), *poac*^*p26ahubb*^ (n = 7) and *ovy*^*p35aluc*^ (n = 5)) and activated (wild type (n = 8), *p33bjta* (n = 5), *poac*^*p26ahubb*^ (n = 4) and *ovy*^*p35aluc*^ (n = 6)) eggs from 3 females per genotype were used in this analysis. Data are means ± SEM. Top graph: *p = 0.0353 and **p = 0.0012 in a nonparametric statistical Kruskal-Wallis test. Bottom graph: *p = 0.0250 in a nonparametric statistical Kruskal-Wallis test, and **p = 0.0011 in a parametric statistical Tukey’s test. **F.** Top row: Animal views of DAPI stained *p33bjta/+* (n = 34, 2 females), and *p33bjta* (n = 36, 2 mutant females) 4 hpf embryos, showing abnormal chromatin organization. Middle row: Lateral views of 4-cell stage embryos from *p33bjta/+* (n = 77/111, N = 2 females), and *p33bjta* (n = 29/59, N = 3) females. Bottom row: Lateral views of 4 hpf embryos from *p33bjta/+* (n = 77/111, N = 2) and *p33bjta* (n = 29/59, N = 3) females. **G.** Top row: Animal views of DAPI stained 4 hpf embryos from *ovy*^*p35aluc/+*^ (n = 19, N = 2), and *ovy*^*p35aluc*^ (n = 9, N = 2 females), showing abnormal chromatin organization. Middle row: Lateral views of 4- and 8-cell stage embryos from two heterozygous (n = 110), and one homozygous (n = 69) *ovy*^*p35aluc*^ females. Bottom row: Lateral views of 4 hpf embryos from 2 heterozygous (n = 110), and one homozygous (n = 10) *ovy*^*p35aluc*^ females. hpf, hours post fertilization. Scale bar = 4 μm (A), 40 μm (C), 210 μm (F, G).

We also investigated whether *p33bjta*, *poac*^*p26ahubb*^ and *ovy*^*p35aluc*^ mutant eggs completed meiosis following egg activation by examining second polar body formation [39]. We found that meiosis resumed normally at 5 minutes post-egg activation (mpa) in the mutants, with sister chromatids evident in anaphase II (Fig 2A). By 10 mpa, an actin associated second polar body was detected in wild-type and mutant eggs (Fig 2A), indicating normal completion of meiosis II.

To address whether other aspects of egg activation were altered in the mutants, we investigated CG exocytosis and chorion elevation. We first defined the egg-to-chorion index (ECI) as a readout of chorion elevation. To calculate ECI, activated egg and chorion areas were measured at 30 mpa (see Materials and Methods). Quantitative analysis of the ECI of *poac*^*p26ahubb*^ eggs showed normal chorion elevation (*p*>0.05) (Fig 2B). However, *p33bjta* and *ovy* eggs displayed a significant, variably expressive reduction in chorion elevation (Figs 1C, 2B, and 2F).

Since chorion elevation is mediated by CG exocytosis, we next examined CG size, area, and number in unactivated eggs, as well as their reduction in activated eggs as an indicator of their exocytosis. We found in wild-type eggs that both small and large CGs were distributed at the cortex before activation with few remaining after egg activation, indicating their exocytosis (Fig 2C–2E). Unactivated eggs of *p33bjta*, *poac*^*p26ahubb*^, and *ovy* tended to have a reduced CG area and *ovy* a reduced CG number compared to wild type, although the difference was not statistically significant (Fig 2D and 2E). At 30 mpa, however, a substantial quantity of CGs and/or CG area remained in *p33bjta*, *poac*^*p26ahubb*^, and *ovy* mutant eggs (Fig 2C–2E), which may cause the variably expressive chorion elevation phenotype. In wild type, we measured a median CG diameter of 22.3 μm and 6.2 μm before and after activation, respectively (Fig 2E, right). In contrast, in *p33bjta* unactivated mutant eggs, the median size of CGs was significantly smaller, 11.4 versus 22.3 μm in wild type. On the other hand, at 30 mpa, the CG average median size in the *ovy*^*p35aluc*^ mutant was 32.7 μm, a value significantly larger than in wild type (Fig 2E). Notably, *poac*^*p26ahubb*^ activated eggs exhibited normal chorion elevation (Fig 2B), while a significant number of small (mean 5.3 μm diameter) CGs persisted (Fig 2E), indicating that the release of the larger ones is sufficient to induce normal chorion elevation.

To examine whether the cytoplasmic defects in oocyte maturation and egg activation impacted embryonic development, we examined the embryos of two of the mutants, *p33bjta* and *ovy* mutant females (Fig 2F and 2G). We found that modest cleavage furrows were evident in some mutant embryos at the 4- and 8-cell stages, however, a blastoderm was not evident at 4 hours post fertilization (hpf). DAPI staining at 4 hpf revealed three classes of blastulae for each mutant compared to wild type, characterized by a single nucleus or pronounced clumping or fragmentation of nuclei at the surface of the opaque yolk, indicating that nuclear divisions proceeded in some embryos but that cytokinesis failed (Fig 2F and 2G). As *blac* eggs degenerate after laying, we did not perform meiosis resumption, egg activation and DAPI staining experiments of this mutant.

These data show that features of egg activation are perturbed in the *p33bjta, poac*^*p26ahubb*^, and *ovy* mutants. While the capacity to undergo meiotic divisions and polar body extrusion is normal, defective cytoplasmic segregation and embryonic progression suggest that specific egg activation traits are regulated by bifurcating or distinct genetic networks.

### *ovy* encodes *adaptor-related protein complex 5*, *mu 1 subunit (Ap5m1)*

**Fig 3:**
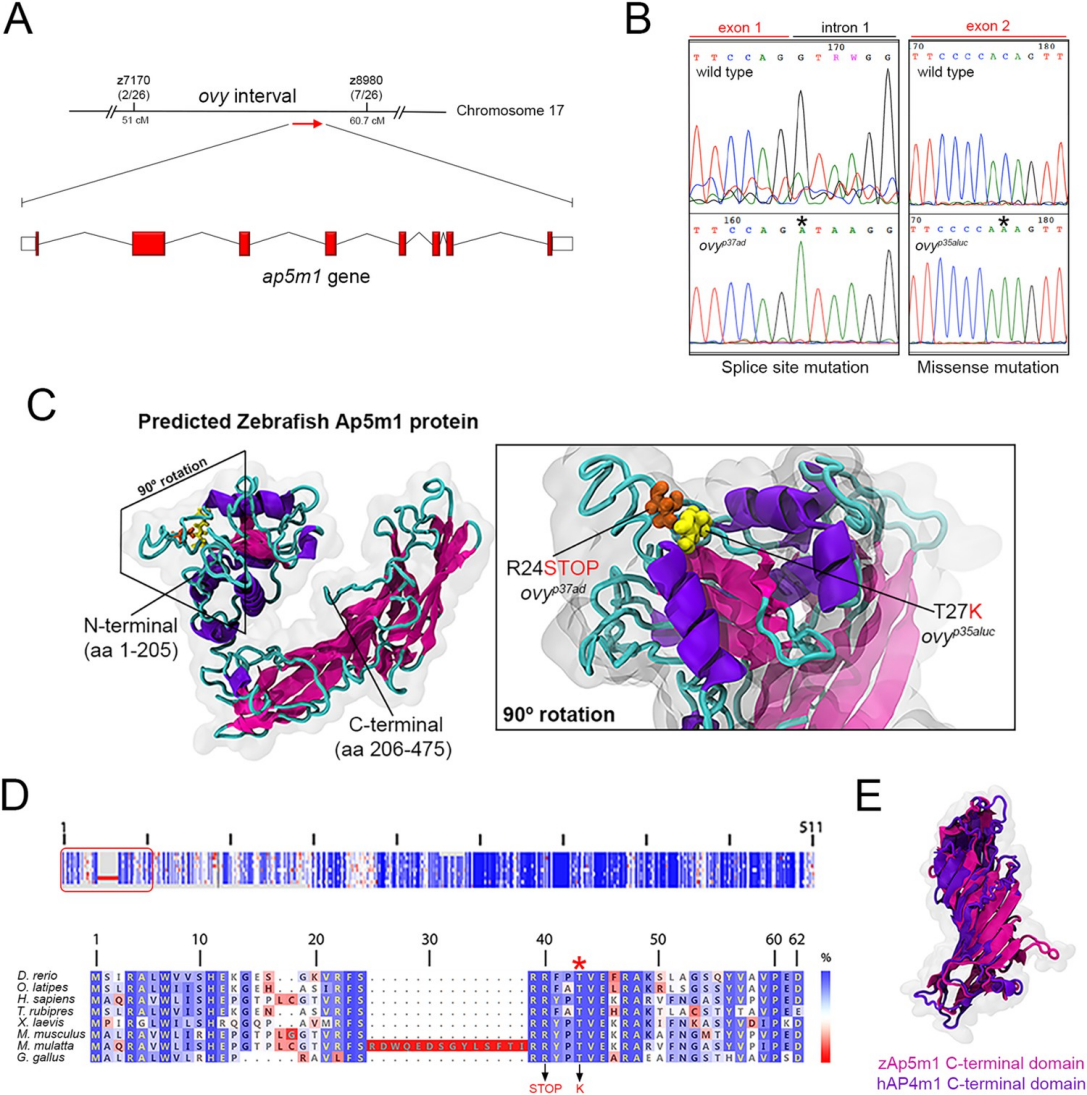
Molecular nature of the *ovy* gene. **A.** Genetic and physical map of the *ovy* locus and schematic representation of the zebrafish *ap5m1* gene, which consists of 8 coding exons and 7 introns. z7170 and z8980 are SSLP markers flanking the *ovy* mutation. In parenthesis, the number of recombinants/total analyzed meioses defining the interval of *ovy*. Exons are shown as red boxes and introns as black lines. Sizes are not to scale. **B.** DNA sequencing analysis of the *ovy*^*p37ad*^ and *ovy*^*p35aluc*^ mutant alleles. Left: genomic DNA sequence of the wild-type and *ovy*^*p37ad*^ allele indicates a single point mutation in the splice donor site of intron 1 of the *ap5m1* gene (red rectangle). Right: cDNA sequence of the wild-type and *p35aluc* allele indicates a single point mutation in exon 2 of the *ap5m1* gene (red rectangle). **C.** Left: predicted tertiary structure of the zebrafish Ap5m1 protein. The α- and β-helixes are colored in purple and pink, respectively, and the connecting loops in cyan. N- and C-terminal domains are indicated. The boundary of the two domains is at residue 205 (arrow head), which is found in the flexible loop comprised by residues 204–208. The approximate volumetric density map of the protein is shown in transparent gray. Right: predicted domain architecture of the Ap5m1 N-terminal portion containing residues Arginine 24 (R24) and Threonine 27 (T27). The amino acid change and premature stop codon caused by the *ovy*^*p35aluc*^ and *ovy*^*p37ad*^ mutations, respectively, are shown. **D.** Multiple amino acid alignments of the Ap5m1 protein and representative members of the vertebrate lineage. Top: Schematic of the protein alignment. The overall percentage (%) identity decreases from top to bottom. The red box indicates the first 49 amino acid residues of Ap5m1. Bottom: Detailed amino acid alignment showing high conservation in fish, amphibians and mammals. Note the high conservation of the Threonine (T) amino acid (red asterisk), which is mutated to Lysine (K) in *ovy*^*p35aluc*^ (indicated in red in the lower panel, along with the splice site mutation, causing a premature stop codon). **E.** Structural superposition between zebrafish Ap5m1 (pink, amino acids 205–475) and the crystal structure of human Ap4m1 C-terminal domains (purple, amino acids 185–453). The RMSD value corresponds to 2.45 Å, thus demonstrating structural identity in overall 3D structure.

To determine the molecular nature of the *over easy* gene, we performed RNA sequencing (RNA-seq) of *ovy*^*p37ad*^ mutant eggs, an approach used previously to identify mutant genes in zebrafish [40–43]. A search of the top ten downregulated genes in the mutant egg transcriptome identified only one gene in the chromosomally defined *ovy* interval on Chr 17, the *a**daptor-related* *p**rotein complex* *5*, *m**u* *1* *subunit* (*ap5m1*) gene (log_2_ fold change = -7.0632) (Fig 3A). It encodes one of four subunits of the fifth Adaptor Protein complex (AP5) [44], the mu or μ subunit. The AP5 heterotetramer complex localizes to a late endosomal/lysosomal compartment and functions in sorting late endosomes back to the Golgi in cultured mammalian cells [44–47].

Next, we amplified the wild-type and mutant *ap5m1* cDNA in three overlapping amplicons to sequence the entire coding region (S2A Fig and S1 Table). The middle and 3’ end overlapping PCR products showed no differences in sequence between wild type and mutant. However, the most 5’ *ap5m1* amplicon amplified successfully from wild type, but was not obtained from mutant cDNA, despite repeated attempts, suggesting a possible splicing defect. Sequencing the 5’ and 3’ ends of intron 1 of *ap5m1* in *ovy*^*p37ad*^ genomic DNA (gDNA) confirmed a splice donor site mutation (G to A) in the exon1-intron 1 junction (Figs 3B and S2B). This results in predicted retention of the first intron in the transcript. Using primers within the first intron and either exon 1 or exon 2, we found that indeed the *ovy*^*p37ad*^ cDNA retained the intron, resulting in a premature stop codon in the intronic sequence (S2B and S2C Fig), expected to cause a loss of all functional domains of the Ap5m1 protein. We then sequenced the *ap5m1* gDNA and cDNA from the second *ovy*^*p35aluc*^ allele and identified a missense mutation (C to A transversion), changing amino acid threonine 27 to lysine (Fig 3B and 3C). This residue is highly conserved in all 9 vertebrate species examined (Fig 3D), suggesting its importance to Ap5m1 function.

To molecularly characterize the Ap5m1 protein, we generated a homology-based structural model of the zebrafish Ap5m1 protein (Fig 3C). Our model indicates that Ap5m1 is comprised of an N-terminal α-helical domain (residues 1–205) and β-sheet structured C-terminal domain (residues 206–492) (Fig 3C). Immunoprecipitation experiments suggest that the N-terminal domain interacts with another subunit of the AP5 complex [48]. The *ovy*^*p35aluc*^ mutation impacts a peptide loop in the N-terminal domain (Fig 3C). The threonine to lysine substitution likely provokes a conformational change that would affect its binding activity [46]. In contrast, the C-terminal or Mu homology domain may be the site of specific cargo recognition and interaction [44]. Importantly, the AP5m1 C-terminal domain showed high conservation with acidic residues of the adaptor protein complex 4 mu4 (AP4m4) subunit C-terminal domain and its overall predicted structure (Fig 3E). AP4m4 is a subunit of the human AP4 complex (another heterotetramer involved in vesicle trafficking), and plays a role in mediating the transport of cargo from the Golgi to endosomes [46,49]; thus suggesting that AP5m1 could function in endosomal trafficking during oogenesis.

The morphological and molecular phenotypic features of the *ovy*/*ap5m1* mutant suggest a functional relationship between the endolysosomal route, YG size acquisition, and yolk protein cleavage during zebrafish oogenesis, which was not known previously in egg-laying organisms. Thus, we provide the first phenotypic study in an animal model system of *ap5m1* gene function.

### Egg activation genes

**Fig 4:**
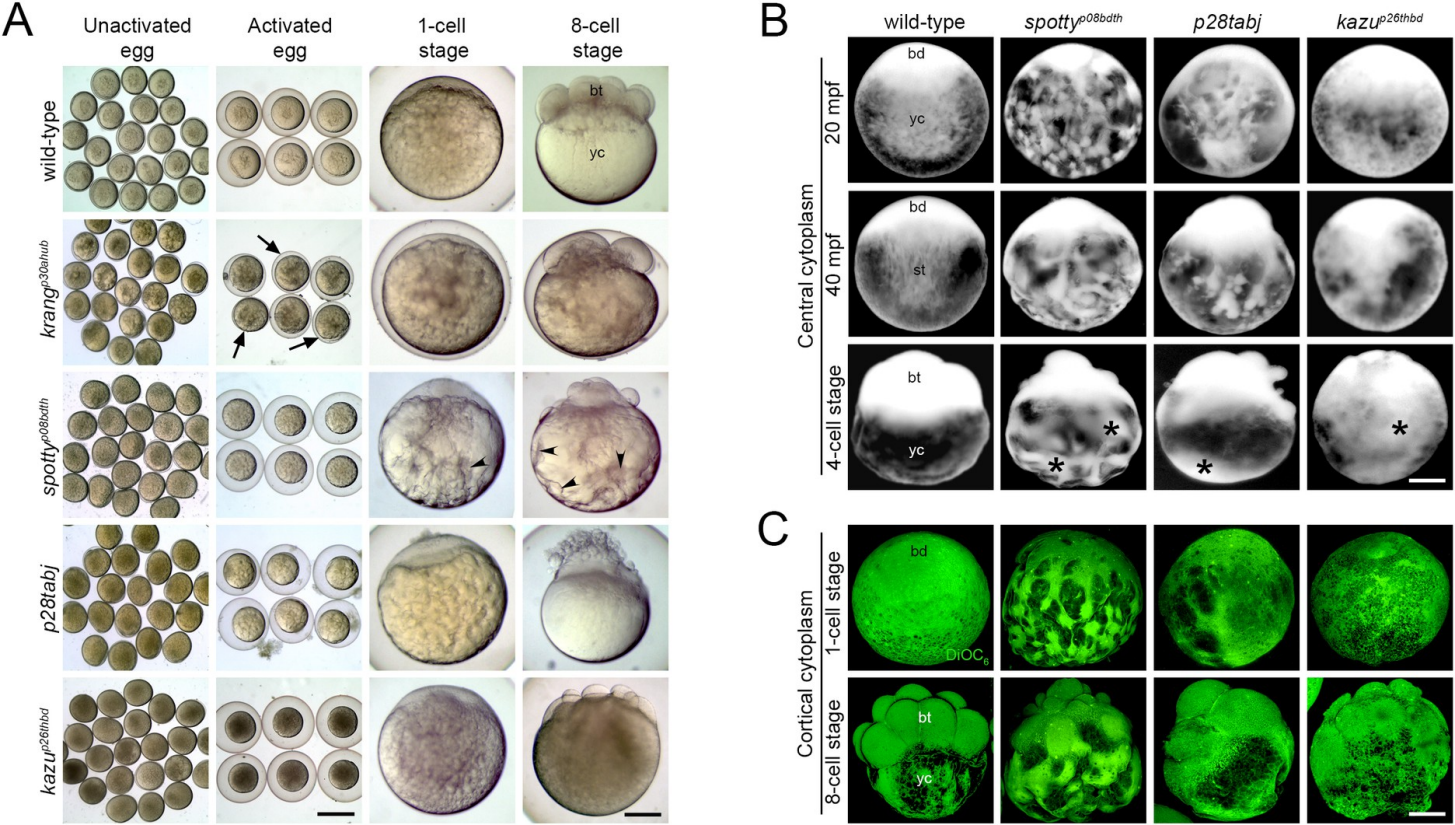
Egg activation mutants. **A.** Bright-field images showing early developmental stages of live wild-type and mutant eggs and embryos. All unactivated eggs collected after gently squeezing gravid females showed no detectable defects. After egg activation, however, the *krang* mutant egg displays a pronounced chorion elevation defect (black arrows). As development proceeds, the cytoplasm is abnormally distributed in the yolk cell (yc) of *spotty* mutant eggs and early embryos (black arrowheads) and a defective blastoderm (bt) is formed. *krang* (1887 eggs from 50 females), *spotty* (1273 eggs from 40 females), and *p28tabj* (2506 eggs from 34 females) and *kazu* (1608 eggs from 32 females). **B.** Lateral view of acid-fixed wild-type and mutant eggs and early embryos showing the organization of the central cytoplasm (bright). In wild type, the blastodisc (bd) grows by animal-ward transport of cytoplasm from the yolk cell (yc) and by the 4-cell stage, the cytoplasm in the yolk cell is no longer evident. In contrast, *spotty*, *p28tabj* and *kazu* mutant eggs exhibited an abnormal distribution and severe retention of cytoplasm in the yolk cell (black asterisks). Wild type (n = 22), *spotty* (n = 17), and *p28tabj* (n = 19) and *kazu* (n = 12). **C.** Lateral view of acid-fixed and DiOC_6_-stained wild-type and mutant eggs and early embryos showing the organization of the cortical cytoplasm (green) and yolk (dark). Wild type (n = 20), *spotty* (n = 20), and *p28tabj* (n = 14) and *kazu* (n = 18). Scale bar = 830 μm (A, columns 1 and 2 from left to right), 150 μm (A, columns 3 and 4 from left to right), 190 μm (B, C).

We identified a second group of four maternal-effect mutants with defects in the egg-to-embryo transition (Table 1): *p30ahub* (henceforth called *krang*^*p30ahub*^, named for a fictional supervillain standing in a confined space), *p08bdth* (hereinafter called *spotty*^*p08bdth*^, named for its spotty cortical pockets of cytoplasm in the yolk cell cortex), *p28tabj*, and *p26thbd* (henceafter called *kazukuram* (*kazu*^*p26thbd*^), which in the Mapuche language, Mapudungun, means gray (kazu) egg (kuram)). We examined two processes of egg activation in these mutants, chorion expansion and cytoplasmic segregation. Immediately after wild-type eggs are laid and prior to their activation, the chorion is closely associated with the egg surface (Fig 4A). Following egg activation, the chorion elevates due to CG exocytosis and the blastodisc forms by the initiation of cytoplasmic transport to the animal pole. We found that the *krang*^*p30ahub*^ mutant showed a severe, highly penetrant small chorion phenotype (Fig 4A). The three other mutants, *spotty*^*p08bdth*^, *p28tabj*, and *kazu*^*p26thbd*^, displayed abnormalities in cytoplasmic subcellular reorganization, subsequent cell cleavage and blastoderm formation, but did not compromise chorion elevation (Fig 4A).

We mapped the *krang*, *spotty*, and *kazu* mutations to chromosomal loci and determined that *p28tabj* was not linked to the intervals of the other mutations reported here or previously (Table 1. See Material and Methods). Based on our phenotypic and mapping analysis, we conclude that these mutations disrupt distinct genes required for chorion elevation, cytoplasmic segregation, and early embryo formation. Importantly, we identified genes that appear to control divergent processes acting in the zebrafish egg-to-embryo transition.

### Cytoplasmic reorganization and blastoderm formation are controlled by maternal egg-to-embryo transition genes

In most animals with telolecithal eggs and discoidal cleavage, including zebrafish, egg activation is characterized by blastodisc elevation, which then forms the blastoderm and ultimately the embryo (reviewed in [50]). During blastodisc growth, the cytoplasm is transported to the animal pole through the process of cytoplasmic segregation in two main phases: the slow (20–30 minutes post fertilization, mpf) and fast (30–40 mpf) flow of cytoplasm [29,32]. The *spotty* and *p28tabj* mutants displayed apparent normal blastodisc formation at 20 and 40 mpf, while *kazu* mutant eggs appeared normal at 20 mpf but displayed a smaller blastodisc at 40 mpf (Fig 4A and 4B). For all three mutants the subsequent cell cleavage stage was morphologically abnormal, with altered cell size and organization, and the *spotty* mutant also displayed cortical pockets of cytoplasm within the yolk cell (Fig 4A and 4C).

To further explore the cytoplasmic segregation process, we visualized the organization of cytoplasmic domains and organelles. In acid-fixed wild-type eggs, the cytoplasm is distributed in the blastodisc and across most of the yolk cell, intermingled with YGs (Fig 4B). As development proceeds, the blastodisc grows by transport of cytoplasm from the yolk cell via specialized transportation pathways or streamers to the animal pole (Fuentes and Fernandez, 2010). The *spotty*, *p28tabj* and *kazu* mutant zygotes showed marked changes in the organization of cytoplasm distributed in the yolk cell during the first phase of cytoplasmic segregation (Fig 4B, 20 mpf). During the fast phase of cytoplasmic segregation when axial streamers form, the distribution of membranous organelles was also affected in the mutant zygotes (Fig 4C, 40 min, 1-cell stage). Cytoplasmic patches in various regions of the yolk cell were present at the 4- and 8-cell stages in these mutants but not wild type (Fig 4B and 4C). Thus, defective cytoplasmic organization and animal-ward streaming, likely explain its retention in the yolk cell in all mutant zygotes studied. This suggests that the mechanisms regulating cytoplasmic segregation are compromised in these mutants.

### Maternal Krang plays a role in cortical granule biology during egg activation

**Fig 5:**
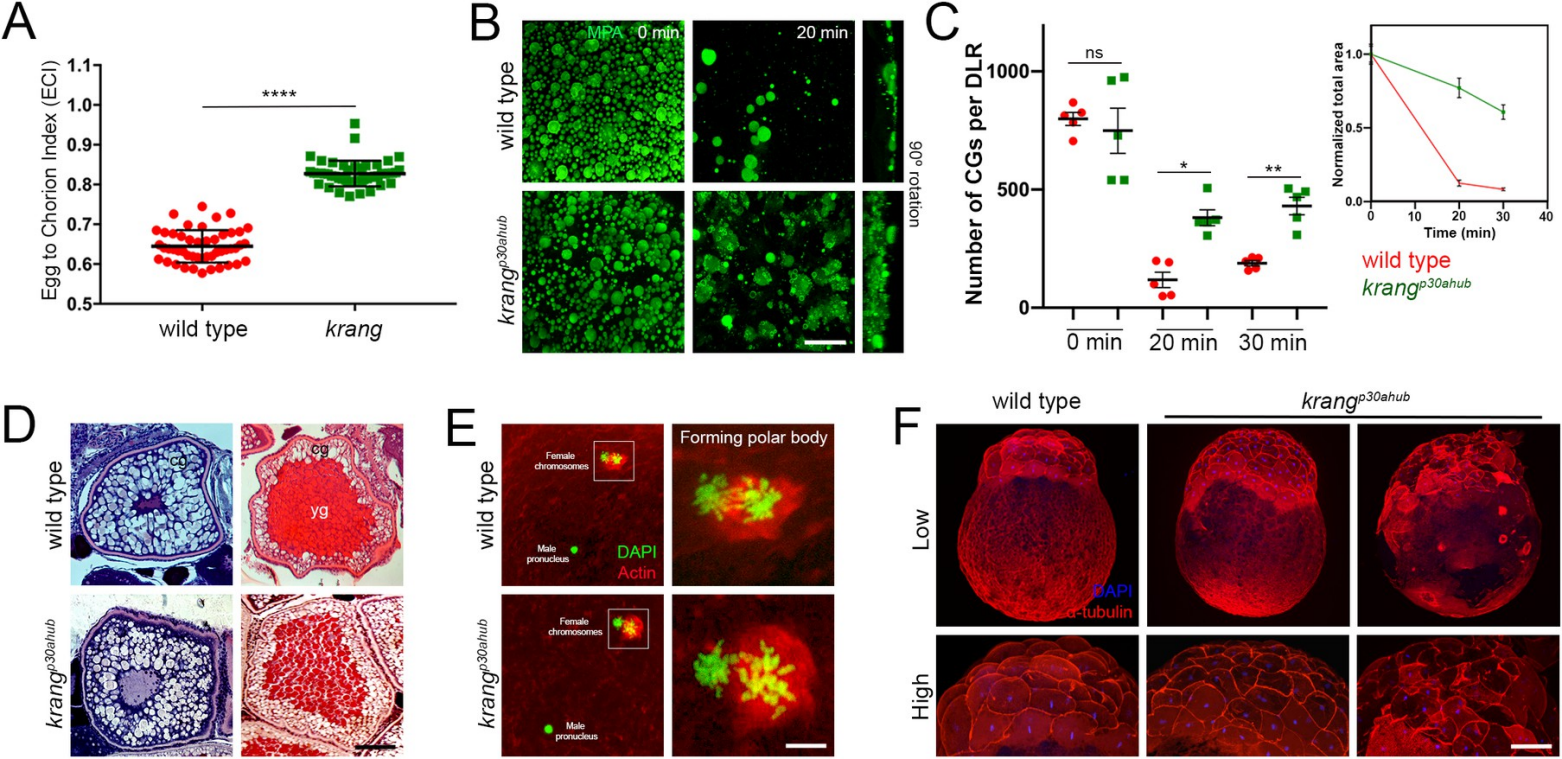
Characterization of the maternal-effect *krang* mutant phenotype. **A.** Quantification of the chorion elevation phenotype. Scatter plots of the measured ECI value show a penetrant chorion elevation defect in the *krang* mutant egg at 30 mpa (ECI = 0.64 and 0.83; SD = 0.006 and 0.005; for the wild-type (n = 48, 2 females) and mutant (n = 48, 2 females) egg, respectively). SD = standard deviation. Data are means ± SD. ****p<0.0001 in statistical clustering analysis in a nonparametric unpaired *t test* of chorion elevation measurements. **B.** Confocal z-projections (5 μm depth) of MPA stained CGs showing their distribution in wild-type (top row, n = 68 eggs from 3 females) and *krang* mutant (bottom row, n = 55 eggs from 3 females) eggs at two different time points after activation. Images were taken in a determined lateral region (DLR) of each activated egg. Notice that smaller, possibly CGs lacking MPA-staining lectin accumulate in the mutant egg at 20 mpf. **C.** Scatter plots of CG exocytosis timing measured in the DLR of the activated egg. The average number of CGs in the *krang* mutant was not reduced compared to wild type at 0 min but is significantly higher at later time points. Normalized total area curves (upper right graph) showing CG exocytosis rate in wild-type and *krang* mutant activated eggs. Five eggs from 3 females per genotype and per time point were used in this analysis. Data are means ± SEM. *p = 0.0131 and **p = 0.0252 in a parametric statistical two-way ANOVA test. ns, not significant. **D.** Hematoxylin and Eosin stained sections of intact stage II (left column) and stage III (right column) oocytes. Wild-type (n = 2 ovaries), and *krang* (n = 2 ovaries) oocytes, were phenotypically comparable. CGs are formed (stage II) and accumulated at the cortex (stage III). **E.** Confocal micrographs of actin- and DAPI-stained fertilized eggs showing normal meiosis completion and second polar body formation (wild type (n = 6, 2 females) and mutant (n = 15, 2 females). **F.** Most *krang* mutant embryos failed to undergo cell divisions. Top row: wild-type embryo with a typical symmetric blastoderm at 2 hpf (left panel, n = 87/93 embryos from 2 females). In contrast, *krang* mutant embryos display relatively normal (middle panel, n = 78/227) or abnormal (right panel, n = 147/227) blastoderm formation (n = 3 mutant females). Bottom row: high magnification confocal micrographs showing the cell architecture in wild-type and mutant blastoderms. cg, cortical granule; yg, yolk globules. Scale bar = 40 μm (B), 25 μm (D, left column), 95 μm (right column), 50 μm (E, left column), 12 μm (E, right column), 160 μm (F, top row), 85 μm (F, bottom row).

The eggs of *krang* females exhibited a fully penetrant small chorion phenotype, as determined by quantitating the extent of chorion elevation (Fig 5A). To investigate whether the pronounced chorion elevation defect in the *krang* mutant results from a defect in CG synthesis, translocation or exocytosis, we performed MPA-staining to specifically label and track CGs. We found that both small and large CGs formed and were cortically distributed in *krang* eggs before activation (Fig 5B). However, at 20 mpa, CGs persisted in the mutant egg, along with numerous small CG-like vesicles showing reduced MPA staining within the vesicle (Figs 5B, 5C, and S3A and S1 Video). By counting the number of CGs per DLR (determined lateral region, see Methods) at different time points after egg activation, we found, as expected, rapid CG exocytosis in wild type, while no such phenomenon occurred in the mutant eggs (Fig 5C). Although unactivated *krang* mutant eggs did not exhibit a significantly reduced count of CGs, the number remaining at multiple time points following egg activation showed a significant difference (Fig 5C). Furthermore, in *krang*^*p30ahub*^ mutants, the rate of CG exocytosis was slower from 0 to 20 mpa (Fig 5C). Quantitative analysis of CG size showed that their mean diameter at 30 mpa is significantly larger in the mutant at 34.3 μm, than in wild type, at 11.9 μm (S3B Fig). In wild type, the median CG size was 10 μm with a similar 11.3 μm median size in *krang* mutants (S3B Fig). These measurements suggest a retention of larger CGs, accompanied by many small CGs, unlike in wild type (Figs 5B, S3A, and S3B).

To explore CG formation and localization during oogenesis, we examined ovaries of homozygous mutant females. We found that oogenesis in *krang* mutants appeared morphologically and histologically normal, where CGs formed in stage II oocytes and then moved and docked to the cell cortex in later oogenesis stages (Fig 5D). Collectively, these results indicate that CGs form and translocate in the *krang*^*p30ahub*^ mutant as in wild type. Possible changes in CG translocation dynamics, content, or exocytosis after egg activation might impact the magnitude of chorion elevation.

We next investigated development of *krang* mutant embryos. We found that *krang*^*p30ahub*^ eggs were fertilized, meiosis II resumed and completed, as revealed by segregating anaphase II chromosomes and second polar body formation (Fig 5E). Also, the initiation of the first mitosis ensued in *krang* mutant zygotes (Figs 5F and S3E). However, during early embryonic development, many *krang*^*p30ahub*^ embryos failed to undergo cleavage or did so abnormally, often dying during blastula stages (Figs 5F and S3C). The *krang*^*p30ahub*^ embryos that survived to 1 dpf exhibited a wild-type phenotype, or a reduced or ventralized body axis (S3C Fig). To test whether defective chorion elevation impairs the cleaving ability of *krang* early embryos, we removed the chorion by pronase treatment after fertilization. Removal of the chorion resulted in an increased number of cleaving mutant embryos that reached the gastrula and 1 dpf stages, mostly displaying a wild type-like phenotype (S3C and S3D Fig).

To investigate whether defects in chorion biogenesis were associated with the chorion elevation defect, we examined its ultrastructure in cross-sectioned wild-type and *krang* whole ovaries by transmission electron microscopy. The chorion is derived from the vitelline envelope, which is made during oogenesis. In the zebrafish, the structure of the vitelline envelope consists of three zones organized horizontally around the oocyte [38,51]. In analyzing the vitelline envelope ultrastructure, we did not find any morphological variation between wild-type and *krang* oocytes (S3F Fig). These results indicate that the chorion elevation defect, but not its biosynthesis, influences the developmental potential of mutant embryos to progress. When removed, it improved cell cleavage and axis formation (S3C and S3D Fig).

### Krang encodes a novel protein

**Fig 6:**
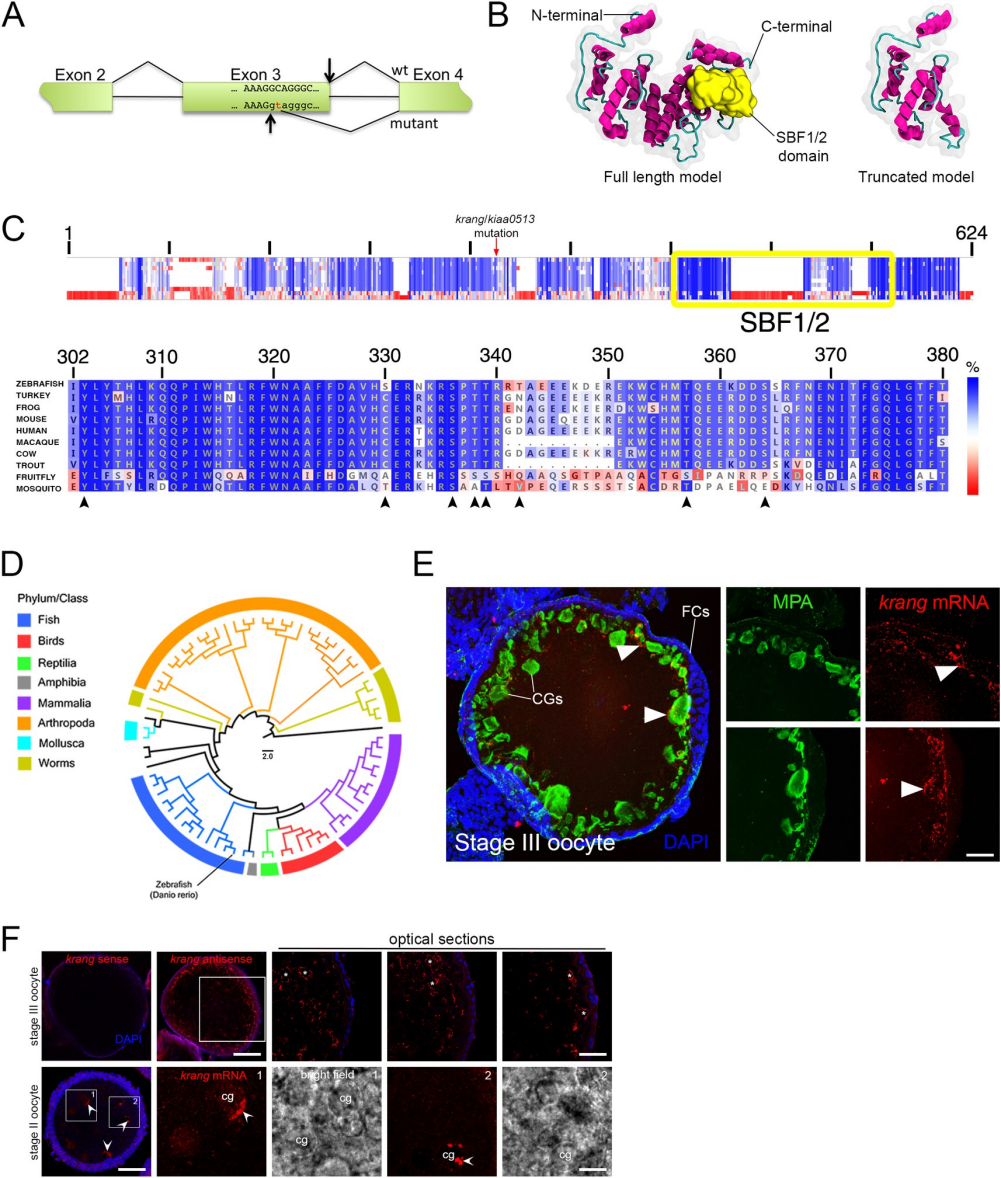
Molecular characterization of the Krang maternal factor. **A.** Schematic representation of the molecular lesion and generation of a premature donor splice site 35 nt upstream of the wild type (wt) donor splice site in the *krang/kiaa0513* (mutant) transcript. **B.** Predicted tertiary structure of the full-length zebrafish Krang protein (left) and its truncated version (right). The α-helixes and the connecting loops are colored in pink and green, respectively. N- and C-terminal domains are indicated. The SBF1/2 functional domain of the protein is shown in yellow. The approximate volumetric density map of the protein is shown in translucent gray. **C.** Krang protein sequence alignment of multiple species represented in E. Top: Schematic representation of the protein sequence alignment. The overall percentage (%) similarity among sequences decreases from top to bottom. The yellow box indicates the SBF1/2 functional domain in Krang. Bottom: Detailed sequence alignment, showing high conservation of this domain in fungi, insects, fish, birds and mammals. Percent similarity is color coded by the scale bar at the right. **D.** Reduced neighbor-joining phylogenetic tree of Krang homologs found in public databases indicating its evolutionary history from invertebrate to vertebrate species. The scale bar in the middle represents evolutionary distances based on residue substitutions per site. **E.** *In situ* hybridization showing the cortically-restricted distribution of *krang* mRNA in a cryosectioned stage III oocyte (n = 18). Notice that the expression of the transcript (arrowheads) is associated with MPA-stained CGs. FCs, follicle cells. Right panels show selected areas (arrowheads) in separate channels. **F.** Whole-mount *in situ* hybridization showing *krang* transcript localization. Top row: wild-type stage III oocyte (n = 43), where *krang* mRNA is peripherally distributed in the cell, presumably overlapping with cortical granules (white asterisks in high magnification images of the same oocyte). Bottom row: stage II oocyte (n = 19), where the *krang* transcript is associated with nascent CGs (bright field and fluorescent high magnification images). Control sense probe did not show transcript signal in stage III oocytes (n = 23, top row, left). Boxes show selected magnified areas. Scale bar = 20 μm (E), 140 μm (F, stage III oocyte), 9 μm (F, top row high magnification images), 55 μm (F, stage II oocyte), 20 μm (F, bottom row high magnification images).

To identify the molecular nature of the *krang* mutant gene, a combination of positional cloning and next generation sequencing strategies was carried out. We mapped the *krang*^*p30ahub*^ mutation to chromosome 18 between markers z65576 and z10008. In testing 1380 females for recombination in the interval, we finely mapped the critical interval of the mutation to 0.87 cM between markers CR925798-1 (38.93 cM) and z58289 (39.8 cM) (Fig 6A). We performed a genomic DNA sequence capture of the entire 1.2 Mb interval from a *krang*^*p30ahub*^ homozygote and wild-type control [52–55], followed by next generation sequencing of the captured DNA. Sequence analysis revealed only 3 single base pair (bp) changes within the physical interval of the mutant gene. Two of the changes were in a large first intron of a gene and the third was in the coding sequence of the same gene. This coding sequence change did not alter the Gly amino acid encoded, but was a wobble codon change from GGC to GGT. None of the changes appeared deleterious, but all were present in the same gene, *kiaa0513*. Thus, we cloned and sequenced the cDNA of *kiaa0513* from *krang*^*p30ahub*^ and wild-type ovaries to determine if it was affected. Interestingly, sequencing of the cDNA revealed a 35 nucleotide (nt) deletion at the 3’-end of exon 3, precisely at the position of the wobble alteration (Fig 6B). Thus, the wobble mutation (C to T) created an earlier splice donor site 35 nts upstream of the wild-type donor splice site (Fig 6A and 6B), generating a transcript lacking 35 nts of the coding sequence.

The *kiaa0513* transcript encodes a 424 amino acid protein with no previously known function, although highly conserved from arthropods to humans (Fig 6C–6E). The 35 nt deletion in the *krang*^*p30ahub*^ cDNA results in a frameshift, which generates a premature stop codon following 24 aberrant amino acids (S3G Fig). As a result, the predicted mutant protein lacks 255 of 424 amino acids, including the highly conserved SET binding factor 1/2 (SBF1/2) or myotubularin-related domain (Fig 6C). SBF1 and SBF2 proteins (Myotubularin-related protein 5 and 13, respectively) have been previously described as homologous to phosphatases but lacking phosphatase activity and instead acting as pseudo-phosphatases regulating the enzymatic activity of other phosphatase myotubularin-related proteins [56–58]. However, the non-catalytic phosphatase domain of these factors was not found in Krang or Kiaa0513 orthologs. Amino acid alignment of zebrafish Krang to other animal orthologs revealed high similarity within the SBF1/2 domain (Fig 6D) and phylogenetic analysis shows the conserved evolutionary relationship of *krang* homologs in vertebrate and invertebrate organisms (Figs 6E and S4). Little is known about Krang/Kiaa0513 to date; therefore, how its SBF1/2 domain physiologically functions in egg activation will need further investigation.

To validate that *krang* is encoded by *kiaa0513*, we generated a second mutant allele, *krang*^*p14del*^, using a CRISPR/Cas9 approach. The new *krang* mutant allele is a 14 bp deletion affecting exon 5, which generates a premature stop codon (S5A Fig). Mutant females also produced eggs that fail to elevate the chorion (S5B Fig). To evaluate if *krang*^*p30ahub*^ and *krang*^*p14del*^ alleles do not complement, we generated trans-heterozygous loss-of-function females (*krang*^*p30ahub*^/*krang*^*p14del*^), whose eggs and early embryos displayed the *krang* chorion elevation and cell cleavage defect (S5B Fig). In addition to the tight chorion phenotype, transheterozygous females yielded ventralized embryos, altogether showing that these two alleles do not complement and are mutations in the same gene, *krang*.

To gain insights into Krang function, we examined its mRNA expression pattern during oogenesis. In previtellogenic stage II oocytes, *krang* transcript puncta were primarily detected around or in nascent CGs, whereas in vitellogenic stage III oocytes, it is peripherally localized in the vicinity of CGs (Fig 6F and 6G). A similar localization pattern has been described for other mRNAs encoding exocytosis and vesicular release proteins in neurons [59]. Taken together, the abnormalities observed in the mutant egg and embryo, and the spatial localization of its mRNA, suggest that Krang functions in regulating crucial components involved in CG dynamics or integrity, which subsequently act in chorion elevation.

### The maternal-effect *kazu* gene controls cell volume acquisition and blastomere adherence during embryogenesis

**Fig 7:**
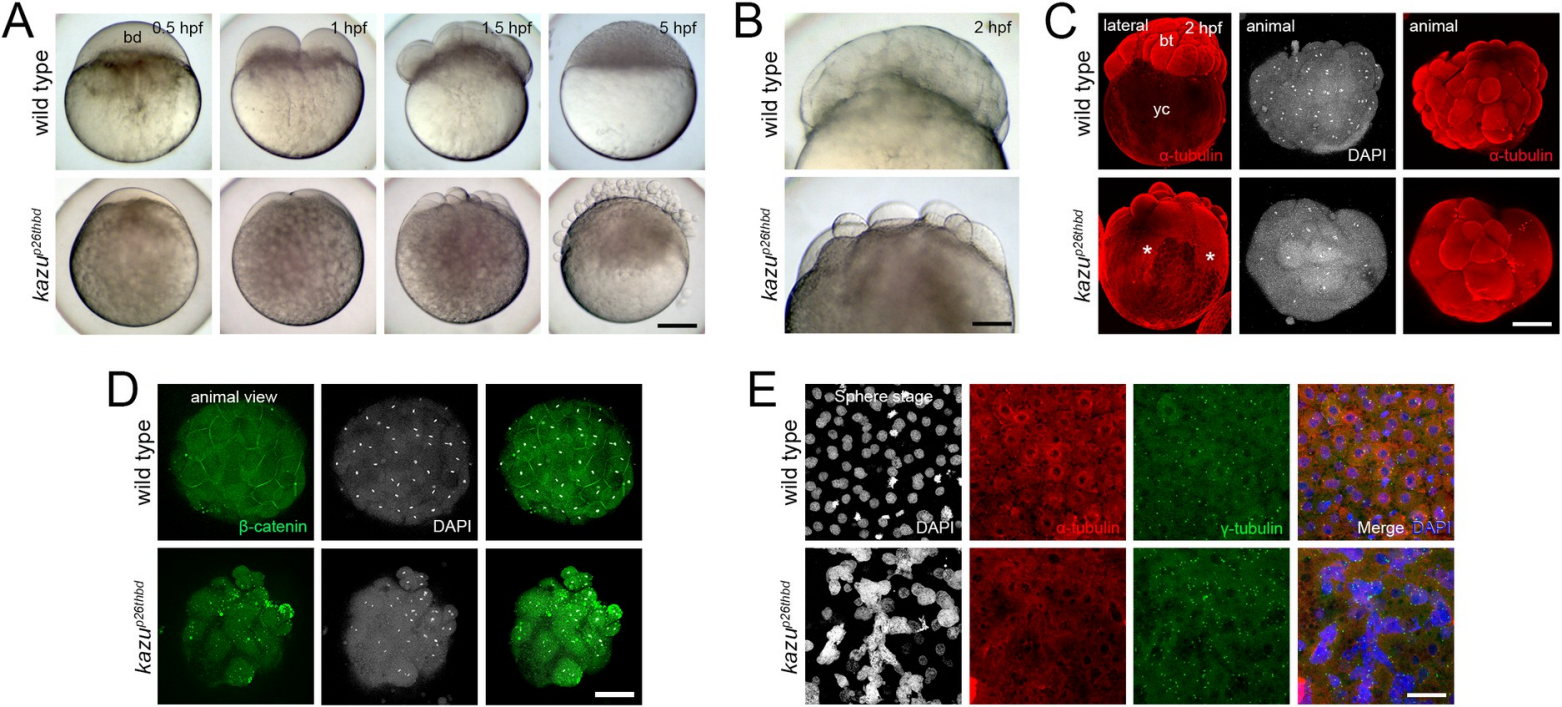
Phenotypic characterization of the *kazu*^*p26thbd*^ mutant early embryo. **A.** Bright field images showing early developmental stages of whole-mount live wild-type (n = 99/99 from 2 females) and *kazu*^*p26thbd*^ (n = 185/265 from 2 females) embryos. The mutant phenotype reveals a smaller blastodisc (bd) with smaller cells during cleavage. These abnormalities in cell size acquisition, together with asynchronous and unequal cell divisions, leads to the formation of a defective blastula with many detached blastomeres. **B.** Higher magnification images showing a cellularized wild-type blastoderm (n = 125/125). In contrast, *kazu*^*p26thbd*^ embryos exhibit a less compacted blastoderm containing smaller cells (n = 62/91). **C.** Lateral and animal views of whole-mount wild-type (n = 31/35) and mutant (n = 39/47) early embryo that show the distribution of α-Tubulin at 2 hpf. In wild-type embryos, α-Tubulin is mainly distributed in the blastoderm (bt) and dividing blastomeres. In *kazu*^*p26thbd*^ mutants, α-Tubulin is distributed in different sized cells and throughout the yolk cell (yc, asterisks). **D.** Animal views of whole-mount wild-type (n = 26) and mutant (n = 8) early embryos that show the localization of β-Catenin at 2 hpf. In wild-type embryos, β-Catenin localized to the blastomere boundaries. In *kazu*^*p26thbd*^ mutants, β-Catenin is aberrantly localized in different cytoplasmic domains. **E.** Animal views of DAPI, α-tubulin, and centrosome staining showing abnormal blastoderm formation at the sphere stage. Cell boundaries and mitotic figures are not observed in most of the mutant (n = 25/33) compared to wild-type (n = 30/35) embryos examined. Notice the formation of a syncytial nuclei layer in the 5 hpf *kazu*^*p26thbd*^ embryo. Scale bar = 180 μm (A), 110 μm (B), 230 μm (C), 165 μm (D), 100 μm (E).

In cleavage stage embryos, the initial cytoplasmic volume at the one-cell stage is cleaved at each cell division in half, with no increase in total cytoplasmic volume until much later in development [60,61]. In zebrafish the extent of cytoplasmic segregation from the yolk into the blastodisc also determines the cleavage stage cell sizes. We found that the eggs of the maternal-effect *kazu*^*p26thbd*^ mutant display a small blastodisc, and the early cell divisions are characterized by asymmetric and irregular cleavage, generating smaller and less compacted blastomeres (Fig 7A and 7B). Additionally, the cytoplasm persisted within the yolk and cell detachment was also observed in *kazu*^*p26thbd*^ cleavage stage mutants (Figs 3B and 7A, 5 hpf). Most *kazu*^*p26thbd*^ mutant embryos failed to develop beyond the sphere stage and died during this period. However, those that survive generally show a reduced body axis (S6A Fig). These results are consistent with a defect in fully segregating the cytoplasm from the yolk cell, with cytoplasm persisting within the yolk at the 4-cell stage and beyond.

We further examined the impact of defective cytoplasmic segregation and a small blastodisc to early embryo development. Immunostained embryos revealed α-Tubulin in mutant embryos extending from the blastomeres into the yolk cell at the 16- to 32-cell stage, whereas it was restricted to the blastomeres in wild-type (Fig 7C). In large parts of the yolk cell, a honeycomb pattern of α-Tubulin was evident, not observed in wild-type, likely reflecting the cytoplasm remaining in the yolk cell that surrounds the yolk globules (Fig 7C). In addition, cell-cell contact was absent in *kazu* early embryos, as revealed by delocalized aggregates of membrane β-Catenin (Fig 7D). In the wild-type sphere stage embryo, microtubules (MTs) and centrosomes were mainly organized around the nucleus or in the mitotic spindle apparatus of well-defined cells (Figs 7E and S6B). In contrast, *kazu*^*p26thbd*^ embryos displayed a severely altered blastoderm morphology at the same developmental stage, revealing a syncytial layer-like blastoderm expanded over the yolk cell, including diffuse MT distribution and multiple centrosomes (Figs 7E and S6B).

These results indicate that Kazu is a critical factor in regulating the incorporation of cytoplasm into the developing blastodisc after egg activation and in the formation of adherent blastomeres cells to form a multi-layer blastoderm during early embryogenesis. Thus, the *kazu*^*p26thbd*^ mutant and other cytoplasmic segregation mutants [13] provide excellent genetic entry points to understand the molecular control of cytoplasmic volume acquisition in blastomere size regulation and in their adherence during early vertebrate embryogenesis.

### *spotty* regulates chromosome integrity, MTOC number, and microtubule nucleating activity during the egg-to-embryo transition

**Fig 8:**
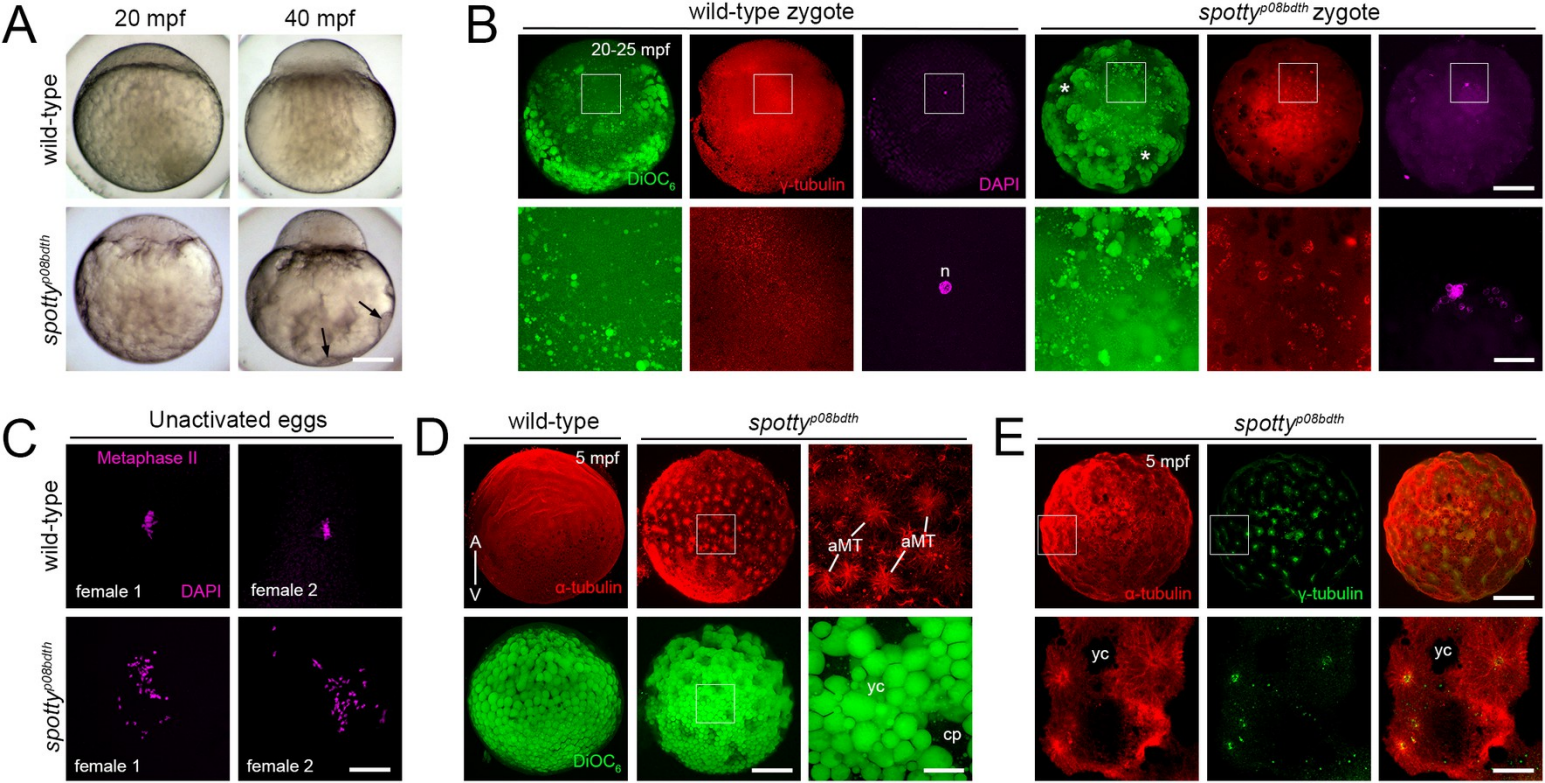
Chromosome, γ-Tubulin and microtubule organization in the *spotty*^*p08bdth*^ mutant egg and zygote. **A.** Ectopic cytoplasmic domain formation in the *spotty* mutant. Instead of a regular organization of cytoplasm intermingled with YGs in the wild-type yolk cell (n = 74 embryos from 2 females), the *spotty* mutant (n = 65 embryos from 2 females) shows numerous, peripheral cytoplasmic domains (black arrows). **B.** Animal viewed confocal images of DiOC_6_- stained zygotes reveal that YG arrangement is affected, coinciding with peripheral patches of cytoplasm (white asterisks). Higher magnification DAPI- and γ-Tubulin-stained images show perturbed nucleus formation and γ-Tubulin assembly in *spotty* (n = 37 from 3 females) compared to wild-type (n = 33 from 3 females) zygotes at 25 mpf. Boxes show magnified areas below. **C.** DAPI-stained wild-type (n = 20 from 2 females) and mutant (n = 48 from 2 females) unactivated eggs showing chromosome organization during metaphase II of meiosis. **D.** α-Tubulin- and DiOC_6_-stained confocal z-projections showing MT and YG distribution at the cortex of wild-type (n = 34) and *spotty* (n = 41) eggs. Higher magnification images reveal the magnitude of such MT structures and YG organization in the mutant egg. **E.** α- and γ-Tubulin co-staining indicates that MTs emanate from numerous MTOCs across the mutant egg (n = 14). Image 4 was selected from a z-stack containing 31 x 1 μm-thick optical sections across the mutant egg. Boxes show magnified (B, D and E) areas. bd, blastodisc; yc, yolk cell; yg, yolk globules; cp, cytoplasmic pocket; A, animal pole; V, vegetal pole; mpf, minutes post fertilization. Scale bar: 190 μm (A), 200 μm (B, top row), 50 μm (B, bottom row), 5 μm (C), 180 μm (D, low magnification), 38 μm (D, high magnification), 180 μm (E, top row), 60 μm (E, bottom row).

Images of live one-cell stage *spotty* showed that at 40 mpf the blastodisc grew after the phase of massive transport of cytoplasm (Fig 8A). However, staining of the yolk cell with DiOC_6_, which labels the YGs due to their high lipid content, revealed multiple, small cytoplasmic domains in cortical regions of the yolk cell between peripheral YGs of the *spotty* mutant, unlike in the wild-type egg (Fig 8B, asterisks). These results point to a cytoplasmic subcellular organization defect in the *spotty* mutant zygote.

During the transition from meiosis to mitosis, the centrosome and astral MT activity is coupled to zygotic nucleus formation and cell-cycle progression [35,39]. The *spotty* mutant shows abnormally cleaved cells (Fig 3A), suggesting altered mitosis. Given that this process is accompanied by γ-tubulin assembly, and the nucleation and organization of MTs, we performed immunofluorescent staining of γ-tubulin and MTs immediately after fertilization. In the wild-type zygote, γ-tubulin is perinuclearly dispersed (Fig 8B). However, the pattern of γ-tubulin distribution was dramatically altered in the mutant zygote with numerous stained aggregates localized around an unusual nuclear architecture in the blastodisc (Fig 8B). Such nuclear organization suggested defective pronuclei behavior and nucleus formation. Fixed DAPI stained wild-type unactivated eggs revealed the establishment of a metaphase plate during meiosis II (Fig 8C). However, several small DAPI spots, possibly dispersed individual or fragmented chromosomes were detected in *spotty* mutant eggs. This suggests that the maternal DNA integrity is compromised by a likely defective meiotic cell cycle in the oocyte. These results reveal that Spotty is essential to: a) regulate or maintain meiosis II metaphase plate integrity and thus chromosome segregation and mononuclear assembly after fertilization, and b) regulate spatially restricted γ-tubulin localization during the first mitotic cell cycle.

In examining MTs in the egg, we found unusual MT arrays in the *spotty* mutant. Multiple unipolar aster-like MT structures were visualized at the cortex at 5 mpf (Fig 8D). Higher magnification optical sections revealed apparent astral-like MT organization (Fig 8D). The localization pattern of γ-tubulin confirmed that these MT-generating structures in the *spotty* mutant were indeed microtubule organizer centers (MTOCs) (Fig 8E). The striking γ- and α-tubulin organization phenotypes in this mutant suggest that the factor encoded by the *spotty* gene regulates MTOC organization, MT architecture and/or dynamics in the egg and zygote.

Altogether, these findings reveal severe defects in MTOC biology in the *spotty* mutant; thus, *spotty* may represent the first described mutation in a vertebrate gene that regulates MTOC number. The identification of the *spotty* gene offers the opportunity to simultaneously understand the mechanism of how MTOCs are regulated in the egg and spatially organized through maternal control during the egg-to-embryo transition.

## Discussion

### Molecular genetics of the oocyte-to-embryo transition

The mutants reported here disrupt genes that are required during oogenesis or activation of the egg. They genetically uncouple nuclear and cytoplasmic aspects of oocyte maturation, and spatially distributed events of egg activation. In addition, they demonstrate that the process of preparing an egg for embryonic development is not a simple linear series of events. Rather, multiple parallel, coordinated events that do not all depend on successful completion of earlier steps in the process. For example, most of the opaque egg mutants can complete nuclear maturation (meiosis) even though cytoplasmic maturation is compromised during oogenesis. The maternal-effect *krang*, *kazu*, *p28tabj* and *spotty* mutants show overtly normal development of the oocyte, including maturation of the cytoplasm and nucleus. However, these mutants do not successfully undergo egg activation. Like opaque egg mutants, the distinct defects observed in these mutants are consistent with oocyte maturation regulated independently of other aspects of egg activation.

The zebrafish has become a useful model system to identify genetic factors controlling human female reproduction and associated diseases (reviewed in [62]). Taking advantage of future genotype-phenotype association studies of oocyte and egg biology in this vertebrate model will enhance our knowledge of the maternal gene bio-network regulating the oocyte-to-embryo transition. Importantly, the accessibility to genetic models will illuminate such maternal factors’ biological function in vertebrate reproduction and development, as well as our understanding of the molecular causes of infertility, and eventually improve clinical applications. However, further studies are needed to fully elucidate the specific functions of these maternal genes and their participation in oocyte maturation, egg activation, and subsequent embryonic development.

### Genetic modulation of YG formation and maturation by egg-laying organisms

The link between YG size acquisition and cleavage of yolk proteins during embryogenesis has been elegantly investigated in invertebrate animals, including insects, starfish and sea urchin [63–67]. However, how YGs form and how yolk protein processing is regulated during oogenesis is still unknown in oviparous vertebrates. Here, we identified four maternal factors controlling YG size and yolk protein cleavage during zebrafish oocyte maturation.

The small YG phenotype in the *ovy*/*ap5m1*, *p33bjta*, and *blac* mutants suggests that their gene products regulate fundamental events in vesicle trafficking and formation, respectively, during and/or after the yolk protein precursor internalization process of vitellogenesis. These studies may provide entry points for future investigations of the molecular control of YG biology [68,69]. Based on the lysosomal nature of YGs and their proteolytic hydrolase content, it is possible that discovering the molecular regulators of YG formation and function may contribute to our understanding of lysosomal related pathologies in humans (reviewed in [10–12]).

Yolk protein cleavage constitutes a critical event to egg-laying species, generating maternal nutrients essential for embryonic growth: the proteolytically cleaved products of Vitellogenin (Vtg), Lipovitellin (Lv) and Phosvitin (Pv) [70]. In addition, antimicrobial activity of Pv and its derived cleavage product has been demonstrated *in vitro* and *in vivo*, including evidence in the immunity of zebrafish embryos [71,72], which opens new avenues for the study of the immunological role of yolk protein cleavage in oviparous animals during oogenesis.

The opaque egg mutant class now comprising 7 mutant genes (here and [13]), all fail in blastodisc formation. Hence, it is possible that the blastodisc defect is a consequence of the failed yolk maturation. However, we cannot exclude that it reflects a secondary function of this class of genes in blastodisc formation. In the former case, two mechanisms could explain the defect in blastodisc formation: i) yolk maturation is a prerequisite for the reorganization of cytoplasm to the animal pole or ii) the persistence of unmodified or immature yolk interferes with blastodisc formation. Despite an initial successful transition from meiosis to mitosis, the progeny of these mutants do not complete development underscoring the requirement for cytoplasmic maturation to produce viable progeny.

Although the molecular identification of regulators of yolk biogenesis and proteolytic processing in multicellular organisms has begun to emerge, much of their functional relevance to embryogenesis remains a puzzle ([13,73–75]; this work). Dissecting the events mediating YG formation and function may help understand: i) conserved or lost functions during oocyte maturation and how different reproductive strategies and nutritional reserves have evolved among vertebrates [7,76], and ii) the temporal and spatial coordination of oocyte maturation and its link to external sources of oocyte-specific signaling molecules including sexual hormones and growth factors in amniotic and non-amniotic vertebrates.

Advances in female gametogenesis, together with the need to decipher molecular phenotypes during this process, may provide new insights into the mechanisms underlying yolk biology prior to oocyte maturation and the contribution to the study of oviparity-to-viviparity transition in metazoans. In this scenario, knowledge of the underlying molecular mechanisms modulating yolk composition and function in egg-laying organisms might also extend previous findings that link the loss of egg yolk genes and the emergence of new genes in the origin of specific traits such as lactation and placentation [7]. Thus, deciphering the molecular circuit controlling yolk biology and function might contribute to understanding the complex cellular energy, nourishment and immunological resource strategies developed in higher organisms during evolution.

### The role of Ap5m1 in reproductive function

The reduced YG size and failure of yolk protein cleavage in the *ovy*/*ap5m1* mutant suggest that Ap5m1 is involved in YG formation and maturation. Ap5m1 might act in the formation of large YGs and in regulating in some manner the activity of YG proteases that cleave Vtg during zebrafish oocyte maturation [68,69]. Our findings and the Ap5m1 molecular pathway provide evidence of a potential role for Ap5m1 in intracellular trafficking and the sorting processes required for YG biogenesis in oviparous organisms.

At the cellular level, the Ap5 complex acts in intracellular trafficking [44–47]. It consists of a heterotetrameric complex including the ζ and β5 (large), μ5 (medium or m5), σ5 (small) subunits [44]. Our molecular modeling of the zebrafish Ap5m1 factor and its homology with the human AP4 complex m4 subunit indicate that it may function in endosomal trafficking during zebrafish oogenesis. The *ovy*^*p35aluc*^ T27K missense mutation is expected to disrupt the domain of Ap5m1 that interacts with the Ap5 β subunit, as observed in *in vitro* studies where that same domain has been mutated [46].

The AP5 complex specifically interacts with proteins SPG11 and SPG15/Spastizin in mammalian cell culture. Mutations in these proteins are implicated in Hereditary Spastic Paraplegia (HSP) disease [46,48]. Intriguingly, siRNA-mediated knockdown of Spg11 and Spg15/Spastizin results in the aggregation of early endosomes, consistent with AP5 functioning with these factors in trafficking in mammalian cells [45]. Each of the five AP complexes localizes to and functions in distinct subcellular endosomal trafficking compartments, with the Ap5 complex shown in cell culture to act at later stages of the endocytic pathway by sorting endosomes back to the Golgi [47]. Our *ovy* mutant represents the first multicellular model for *ap5m1* gene function and concomitantly, the Ap5 complex, providing new avenues to study the oogenesis endolysosomal-YG route in a possible vertebrate model of HSP.

Interestingly, a role for Spg15/Spastizin in reproduction has been reported in zebrafish [73]. The maternally deposited Spastizin factor regulates cortical granule secretory granule maturation and like the *ovy*/*ap5m1* mutant the *spastizin* mutant also displays a similar opaque egg phenotype and a defect in yolk protein processing [13,73]. Therefore, it will be interesting in future studies to investigate how Ap5m1 and Spastizin association functions in YG biogenesis and major yolk protein processing.

In this work, we identified a new regulator, the *ap5m1* gene, as a valuable genetic tool for the study of lysosome-related organelles and its function in the endolysosomal pathway in development. Indeed, our results indicate a novel role for Ap5 complex function in female reproduction. Sorting of Cathepsins and ATPases into lysosomes is essential for yolk protein cleavage [68,69]. In fact, the role of maternal Arl8b protein in Cathepsin-L sorting and lysosomal-mediated degradation of maternal factors in visceral yolk sac has been described in mammalian embryos [77].

Our results highlight the importance of endolysosomal trafficking and protein cleavage during vertebrate embryogenesis. We hypothesize that YG biogenesis relies on Ap5 complex function and its interaction with Spastizin. Epistasis analysis and biochemical approaches involving the sorting of proteases and electrogenic pumps into lysosomes might help elucidate Ap5m1-mediated function during oogenesis.

### Spatial and temporal control of maternal gene function in early embryogenesis

The *kazu*, *p28tabj*, and *spotty* mutant phenotypes show that while chorion elevation and blastodisc formation are both triggered by egg activation, they can be genetically separated and are linked to the formation of compartmentalized cytoplasmic domains. This supports a model in which regionalized and distinct or divergent pathways in the egg regulate these aspects of egg activation. While separable genetically, other maternal-effect mutants show defects encompassing all or many aspects of egg activation, reflecting components acting in early steps of egg activation [20,78,79]. For example, *brom bones/ptbp1a* is defective in inositol 1,4,5-trisphosphate (IP_3_) production and the Ca^2+^ wave that triggers egg activation, thus affecting all aspects of egg activation [20]. The maternal-zygotic *dachsous 1b* mutant exhibits slower activation of the egg, chorion elevation, cell cleavage, and early development [79]. The nature by which this atypical cadherin regulates egg activation processes and early embryonic development broadly is unclear. The maternal-effect *aura/mid1ip1l* mutant alters only a subset of egg activation processes with its primary function in regulating the cytoskeleton [78]. Hence, downstream of Ptbp1a and IP_3_, Dachsous and Aura, likely act in divergent egg activation pathways. Our findings of mutants affecting either CG biology or cytoplasmic segregation further show that egg activation-associated events are regulated independently.

### *Krang*: An uncharacterized and conserved regulator of cortical granule biology

Following exocytosis, the contents of CGs modify the vitelline envelope causing it to expand and enlarging the perivitelline space, which is a well-characterized phenotypic trait to analyze high-quality eggs in assisted reproductive technology (ART) [27,28,80–82]. In addition, mutants with compromised CG exocytosis or vitelline envelope formation generate eggs with a smaller perivitelline space compared to wild type [20,51,73,78] or delayed enlargement of the space [79]. Therefore, insight into the genetic program governing CG biology and vitelline envelope formation may provide biological markers for evaluating oocyte and egg quality in human ART. Recent findings have revealed new insight into the regulation of CG maturation and exocytosis during the vertebrate egg-to-embryo transition [20,28,73,81,83,84], this work). However, a more comprehensive understanding of the molecular basis of how key maternal factors regulate CG biogenesis, dynamics and content release remains to be revealed.

The loss of Krang function impairs egg activation, specifically in chorion elevation. We do not know the nature of Krang’s function in modulating secretory granule biology during female reproduction. Clues for its function are revealed by *krang* transcript localization, which is closely associated with CGs, strongly supporting a spatially restricted function for this factor to CGs. These findings might shed light on a maternal post-transcriptional regulation machinery that localizes translation, possibly to facilitate protein localization and function to, or within, CGs to regulate their translocation, content or function during egg activation. To understand Krang’s function in CG biology will require further investigation.

The human ortholog of Krang, KIAA0513 (71% identity), has been predicted to function during neural development and its transcript to be dysregulated in individuals with schizophrenia and Alzheimer’s Disease [85,86]. The *krang*/*kiaa0513* transcript is also zygotically expressed in the zebrafish hatching gland and nervous system (Thisse and Thisse, 2004). Indeed, the identification of KIAA0513 splicing variants in the brain of related vertebrates, including the zebrafish, has also brought to light a possible exclusive and tissue-specific function of Krang in neural development [85,86]. If Krang acts in an oocyte-specific manner in vertebrates remains to be explored.

Our data strongly support a novel role of Krang during the zebrafish egg-to-embryo transition, presumably modulating its function by specific subcellular localization. Importantly, as this factor constitutes a biological marker of schizophrenia and AD in humans, insights into Krang function might contribute to advancing our current understanding for the still obscure cellular etiology of these neuropathologies and their treatments.

### Maternal determinants of cytoplasmic organization during early embryogenesis

Following fertilization, broadly in animals, early embryonic cells divide and maintain the total cytoplasmic volume inherited prior to the first cell cycle. These early cells are also unusually large, and subcellular structures, such as the nucleus and mitotic spindle, scale to a large extent to the larger cell size [87–91]. Despite these findings, how the total volume of cytoplasm is regulated and distributed, remains poorly understood. We identified the *kazu*^*p26thbd*^ mutant, which displays defects in blastodisc volume acquisition and small cells, thus representing a regulator of cytoplasmic domain formation during early embryogenesis.

After egg activation, a secondary and slower calcium transient observed in the central egg region coincides with and signals the spatial reorganization and animal-directed flow of cytoplasm [21,29]. It is possible that Kazu acts in blastodisc size acquisition by regulating the rate of the animal-ward cytoplasmic flow from the yolk cell, possibly in response to a calcium signal to appropriately allocate the subcellular yolk cell cytoplasmic domains to the zygote (reviewed in [50]). Actin, MTs, molecular motors and their regulators have been implicated in cytoplasmic segregation and RNA translocation through inhibitor and genetic studies [19,29,32,33,79,92,93]. Therefore, it is possible that *kazu*^*p26thbd*^ and other cytoplasmic segregation mutants disrupt upstream regulators or downstream targets of Actin/Myosin contractility and/or MT dynamics during egg activation [19,29,32,33,93–96]. Ultimately, these cytoplasmic transportation defects may affect the arrival of maternal determinants to the developing blastodisc [29,94,95]. Future transcript localization analysis in *kazu*^*p26thbd*^, and other cytoplasmic segregation mutants, would test this hypothesis.

Cell survival critically depends on cell volume regulatory mechanisms. Impaired steady-state cell volume regulation underlies physiological and pathological abnormalities such as cancer and hypertrophy (reviewed in [97–99]). In zebrafish, mutant genes that result in small blastodisc and blastomere phenotypes are candidates for acting in cytoplasmic domain formation and cell volume (reviewed in [50]). Thus, the *kazu*^*p26thbd*^ mutant provides the opportunity to study the maternal control of spatial distribution of embryonic cytoplasm and cell volume acquisition, which may also have relevance in disease.

### Maternally-controlled MTOC assembly and microtubule nucleating activity during early embryogenesis

In most animal cells, the centrosome orchestrates the nucleation of both cytoplasmic MTs and the mitotic spindle during interphase and mitosis, respectively [100–103]. During meiosis in most animals, however, an acentrosomal MTOC (aMTOC) regulates the meiotic divisions, due to elimination of the maternal centrosome during oogenesis. Despite their importance as MTOCs, the genetic regulation of many aspects of MTOC and aMTOC assembly, structure, composition, and function remain elusive. Intriguingly, multiple MTOCs/centrosomes are a hallmark in a growing list of human cancers, emphasizing the importance of regulating MTOC properties and number in health and disease [104–106]. Our understanding of how MTOCs acquire the ability to control their copy number and orchestrate the nucleation of cytoplasmic MTs, impacting on genome stability and embryonic progression, is poorly understood.

During meiosis and early embryogenesis, little is known about the genetic program controlling how the oocyte, egg, and early embryo: a) transitions from centriolar to an acentriolar MTOC, and then back to a centriolar MTOC in the embryo, and b) how they keep MTOC *de novo* biogenesis repressed. This may be a particularly challenging task in the egg, which is chock full of the factors needed to generate the multitude of centrosomes and MTs that function during early cleavage divisions prior to zygotic genome activation. There are few known regulators of centrosome elimination and copy number control. In *Drosophila*, centrosome elimination is determined by Polo kinase activity and the reduction of pericentriolar material (PCM) components, which causes centriole loss before meiosis initiates [107]. We do not know if the ectopic MTOCs in *spotty* represent centrosomal or acentrosomal MTOCs.

The excessive number of aster-like MT structures with γ-Tubulin aggregates in the *spotty* mutant egg suggests that Spotty functions in repressing MTOC number and MT organization during the egg-to-embryo transition. Additionally, the temporal, geometric and spatial organization of MTs and γ-Tubulin in the *spotty* mutant reveal two important features of its genetic product and the mechanisms behind its activity. First, in addition to the recruitment of γ-Tubulin and other MTOC components, the gene product may be necessary for assembling and positioning the functional MTOCs after fertilization. Second, it may be critical for MT dynamics by negatively controlling the nucleating activity of the MTOC. Determining the molecular identity of the *spotty* gene is expected to identify a new repressor of supernumerary MTOCs and MT assembly during the oocyte-to-embryo transition. However, the origin of the defect in *spotty* mutant will require further investigation during oogenesis. Here, we present a genetic and functional model for the *in vivo* study of MTOC copy number regulation and MT architecture control during early vertebrate embryogenesis.

## Materials and methods

### Ethics statement

All animal protocols were approved by the University of Pennsylvania Institutional Animal Care and Use Committee (IACUC) and University of Concepcion Institutional Animal Care (CEBB 818–2020).

### ENU mutagenesis and fish husbandry

TÜ and AB strain males were mutagenized with 3.3 mM ENU as described in [108]. The mutagenesis efficiency was determined by crossing mutagenized males to females that were triple mutant for three mutations that disrupt pigment formation, *albino* (0.04%), *golde*n (0.12%) and *sparse* (0.12%) as in [108]. Through the natural mating strategy, we generated 503 F3-families, each bearing two independently mutagenized genomes. The 716 genomes screened for maternal-effect mutations was calculated according to the formula for pooled F3 embryos as previously described [13].

Female maternal-effect mutants were identified by crossing F3 females to sibling/cousin or wild-type males and examining their F4 progeny. Phenotyping analysis was performed immediately after egg collection for fertilization and egg activation morphological defects and later at 1-day post fertilization for morphological defects and viability. Females that produced greater than ~40% phenotypic progeny were retested in crosses with wild-type males to confirm that the phenotype was a strict maternal-effect. No mutants exhibited maternal-zygotic genetics. For further phenotypic characterization and mapping, female maternal-effect mutants that reproducibly produced phenotypic offspring when crossed with a wild-type male were recovered typically from 10–15 intercrosses of the F2 generation of each family. Adult wild-type and mutant fish were maintained for a 14 hr light/10 hr dark photoperiod at 28°C.

### Genome mapping, and sequencing for *ovy*/*ap5m1* and *krang*/*kiaa0513* genes identification

The oocyte-to-egg transition mutations were mapped to a chromosomal locus as described [13,109,110]. Positional mapping and cloning were carried out through bulk segregant analysis using SSLP markers [111]. The *ovy*^*p35aluc*^ and *poac*^*p26ahubb*^ mutations were both mapped to Chr 17 but to distinct genetic intervals between 51 and 61 cM, and 58 and 79 cM, respectively (Table 1). By testing 26 females for recombination, the *ovy*^*p35aluc*^ interval was defined to an interval of 0.7 cM between markers z7170 (2/26 recombinants) and z8980 (7/26 recombinants). Linkage analysis of *p35aluc* and *ovy*^*p37ad*^ mutations located them to the same interval on chromosome (Chr) 17. The *ap5m1* gene exon 1-intron 1 and intron 1-exon 2 junctions were amplified by PCR from *ovy*^*p37ad*^ gDNA using primers targeting the 5’ and 3’ ends of intron 1 (S1 Table). The *ap5m1* missense mutation was identified by sequencing the first overlapping gene amplicon from the *p35aluc* mutant background using specific primers (S1 Table). The *blac*^*p25bdth*^ mutation was mapped first to Chr 5 and then the *blac*^*p26ahubg*^ allele was mapped to the same locus on Chr 5 between 47 and 64 cM (Table 1).

The *krang*^*p30ahub*^, *spotty*^*p08bdth*^, and *kazu*^*p26thbd*^ mutations were mapped to chromosome (Chr) 18 (between 33 and 47 cM), Chr 2 (between 44 and 62 cM), and Chr 8 (between 95 and 104 cM), respectively (Table 1). For the *krang*^*p30ahub*^ mutation, the CR925798-1 marker was generated for fine mapping (see S1 Table). Chromosomal locations of the SSLP markers were determined using the zebrafish reference genome Zv9.

For next generation sequencing, enriched and captured sequences (~800 kb) were extracted from gDNA of wild-type and *krang*^*p30ahub*^ females as previously described [54]. The libraries were then sequenced using an Illumina Genome Analyzer II.

### Generation of *krang*^*p14del*^ mutant

Genome editing was carried out by targeting *krang*/*kiaa0513* in exon 5. A CRISPR target site 5′-gattgacagATGTCACCAGGTGG-3′ in intron 4 and the fifth coding exon of *krang*/*kiaa0513* was selected. The sgRNA construct was purchased from the University of Utah Mutation Generation and Detection Core. For injection, the synthetized sgRNA was incubated with Cas9 protein (PNA Bio, Cat# CP02-250) to form an RNP complex. 1–3 nl of 2.5 μM RNP complex was injected into 1-cell stage embryos. To evaluate cutting efficiency, we performed HRM (High Resolution Melt assay) analysis on F0 single injected embryos using MeltDoctor HRM Master Mix (Applied Bio-systems). Optimized flanking primers for HRM are indicated in S1 Table. Remaining injected embryos were raised to adulthood to produce F1 families. To identify the *krang*/*kiaa0513* molecular lesion, we sequenced the amplified PCR product from transheterozygous fish genomic DNA using primers flanking the CRISPR/Cas9 target site (Exon 4-Intron 5, S1 Table).

### Gene complementation and PCR-based genotyping

Complementation tests were performed by crossing two *poac*^*p26ahubb*^ heterozygous females to two homozygous *p35aluc* males and two heterozygous *p35aluc* females to two *ovy*^*p37ad*^ homozygous males and raising the progeny to adulthood. Transheterozygous *p35aluc/ovy*^*p37ad*^ females produced 100% mutant opaque eggs, whereas transheterozygous *p35aluc/poac*^*p26ahubb*^ females produced all wild-type embryos (36/36 and 38/38 females, respectively). Genetic complementation analysis between the two *blac* mutations was conducted crossing three *p26ahubg* heterozygous females to either *p25bdth* homozygous males (n = 2) or a heterozygous male (n = 1). Fish were raised to adults and females tested for the lysed egg phenotype. These mutations failed to complement each other, generating transheterozygous mutant females from all 3 crosses in the expected ratios (10/20, 15/31, and 2/10 females for the three respective crosses). Also, a *krang*^*p14del*^ heterozygous female was crossed to a *krang*^*p30ahub*^ homozygous male to generate transheterozygous adult fish. Five transheterozygous and sibling *krang*^*p30ahub*^ heterozygous females each were tested for phenotype. All transheterozygote females produced mutant embryos with small chorions, whereas sibling heterozygotes produced wild-type embryos. All transheterozygous females were tested at least twice and up to 5 times, reproducibly showing the mutant phenotype.

*poac*^*p26ahubb*^, *blac*^*p25bdth*^, *blac*^*p26ahubg*^, and *kazu*^*p26thbd*^ alleles were genotyped using flanking SSLPs designed for positional cloning. *ovy*^*p35aluc*^, *ovy*^*p37ad*^ and *krang*^*p30ahub*^ alleles were genotyped using KBiosciences Competitive Allele-Specific PCR genotyping system (KASP, KBiosciences) (see S1 Table).

### Whole-mount fluorescent *in situ* hybridization and CG staining

Fluorescent *in situ* hybridization of *cycB1* [112], *dazl* [113] and *krang* (this work) was performed in whole-mounted oocytes and cryosectioned ovaries (30 μm thick). Oocytes were isolated and fixed according to [114]. For staining of frozen tissue, ovaries were dissected, fixed, and stored in 30% sucrose as described in [115]. Isolated and cryosectioned oocytes were pre-hybridized, hybridized, washed and blocked as described in [114]. Samples were then incubated in the Anti-POD antibody (1:500; Roche) and washed 4 times for 25 min in 1X PBS. For fluorescent mRNA detection, samples were developed in TMR-Tyramide Signal Amplification (TSA) system in staining buffer (1:50, PerkinElmer), 10 min at room temperature (RT) in the dark. After 3 times washing for 5 min in 1X PBS, samples were incubated in *Maclura pomifera* Agglutinin (MPA) diluted to 50 μg/ml as described in [116,117] for 20 min in the dark at RT. Then washed in 1X PBS 4 times for 5 min each and mounted in Vectashield mounting medium with DAPI. The *krang* digoxigenin-labeled probe was synthesized *in vitro* using DIG RNA labeling kit (Roche) with T3 (sense) or T7 (antisense) polymerase from *kiaa0513* full length DNA sequence cloned into the pBluescript II SK+ vector.

For CG staining, unactivated and activated eggs were collected from gravid females by squeezing and by natural crosses, respectively. Unactivated eggs were kept as described [20], and activated in a hypotonic solution of 1X E3 buffer. Eggs were then acid-fixed at 0, 5, 20 and 30 mpa intervals in 5% formaldehyde and 5–8% glacial acetic acid at room temperature, manually dechorionated and placed in gently agitation for 2 hrs, washed in 1X Phosphate-Buffered Saline (PBS) 4 times for 10 min each [118]. Samples were labeled with fluorescent conjugated MPA for 30 min as described above. Then washed in 1X PBS 4 times for 10 min each and either placed in small petri dishes for *in toto* observation or mounted on glass slides.

### F-actin and meiotic chromosomes labeling

For actin labeling, zygotes were fixed, washed and blocked as in [117]. Then, TRITC-Phalloidin (Sigma) was diluted to 0.5 μg/ml in PBT. Samples were mounted in Vectashield mounting medium with DAPI on glass slides.

### Histology

Ovaries dissected from euthanized females were fixed overnight at 4°C in 4% paraformaldehyde. The next day the fixed tissue was washed in PBS and dehydrated in MeOH. Ovaries were embedded in JB-4 Plus plastic resin (Polysciences) and 5–10 μm sections were cut using a Leica RM 2155 microtome. Sectioned ovaries were stained with Hematoxylin (Sigma-Aldrich), washed in distilled water, stained in Eosin Y (Polysciences), washed in distilled water, and cleared with 50% EtOH. Stained sections were coated with Permount (Fisher), and coverslipped.

### SDS-PAGE analysis of major yolk proteins

Ovaries were dissected and sorted oocytes were obtained as described in [13]. Sorted oocytes and eggs were homogenized in sodium dodecyl sulfate (SDS) buffer with bromophenol blue and glycerol. Samples were heated at 100°C for 5 minutes, vortexed, centrifuged, and proteins were resolved on 8% or 12% acrylamide gels and visualized with Coomassie blue.

### YG diameter, ECI, and CG number and dimensions measurements

For YG size quantification, the mean diameters of YGs from wild-type and mutant stage III oocytes were measured on micrographs taken in whole sectioned and stained ovaries (n = 2 wild-type and 2 mutant ovaries). 3 different cytoplasmic regions (DCRs) of 96 x 96 μm (height x width) within the same wild-type (n = 12 total DCRs from 4 oocytes) and mutant (n = 6 total DCRs per allele from 2 mutant oocytes) oocyte were determined to calculate the individual YG diameters.

To quantitatively examine chorion elevation, the surface area of the egg or chorion was measured. Then, the ratio of the chorion relative to the wild-type 30 mpa egg, which was defined as the egg-to-chorion index (ECI), was determined. The ECI for wild-type eggs was defined as the numerical value for normal chorion elevation (Figs 2B and 5A).

Wild-type and *p33bjta*, *poac*^*p26ahubb*^, *ovy*^*p35aluc*^ and *krang*^*p30ahub*^ CGs were counted in a determined lateral region (DLR) of 60 x 212 x 212 μm (depth x height x width) in egg Z-stacks at 0, 20 and 30 mpa from 2 wild-type and 2 mutant females. To automate the assessment of CG number and dimensions, Z-stacks underwent denoising using the PURE-LET algorithm (https://doi.org/10.1016/j.sigpro.2009.07.009) with the maximum cycle count. Subsequently, background and shading corrections were executed across the stacks using the BaSiC tool [119]. Each Z-stack was transformed into a 2D image via a maximum projection in Fiji [120]. Individual CGs were then pinpointed using the deep learning-driven StarDist method (https://doi.org/10.1007/978-3-030-00934-2_30, https://doi.org/10.1109/WACV45572.2020.9093435), specifically employing the "Versatile (fluorescent nuclei)" built-in model within Fiji. Any segmentation errors in the images were rectified manually.

Prism 7 (GraphPad) software was used to make graphs and for statistical analysis of the data. Samples sizes and statistical values are shown in corresponding figure images or legends. Data are represented as mean ± SD or SEM.

### Cytoplasmic domains visualization

Organization and distribution of cytoplasmic domains were examined under a Leica MZ 12.5 stereomicroscope after acid-fixation of wild-type, *krang*, *kazu*, *p28tbja*, and *spotty* zygotes and early embryos as described [29,118]. Central and cortical cytoplasmic domains were transformed from dark into whitish cytoplasm, and from transparent into dark yolk by inverting the contrast using the Adobe Photoshop CC 2017 software. Zygotes and early embryos were incubated in DiOC_6_ (5 μg/ml), which following acid-fixation marks central and cortical cytoplasmic domain organization. DiOC_6_-stained images were obtained using a Zeiss LSM 880 confocal microscope and the Plan-Apochromat 10×/0.45.

### Whole-mount immuno- and actin staining

For microtubule and actin labeling, eggs, zygotes, and early embryos were fixed, washed and blocked as in [121] and [117], respectively. For staining of cryosectioned tissue, ovaries were dissected, fixed, and stored in 30% sucrose as described [115]. Primary antibodies: mouse anti-α-tubulin (1:500, Sigma) and rabbit anti-γ-tubulin (1:2000, Sigma). Secondary antibodies: anti-mouse IgG1, or anti-rabbit IgG, Alexa 488, Alexa 594, Alexa 633 (All 1:1000, Molecular Probes). When needed, samples were counterstained with DiOC_6_ (5 μg/ml), then washed in 1X PBS 4 times for 10 min each and mounted in Vectashield mounting medium with or without DAPI on glass slides.

### Pronase treatment

Fertilized wild-type and *krang*^*30ahub*^ eggs from 2 females each were treated with 0.25 mg/ml pronase solution in 1X E3 buffer for 10 minutes at 28°C. Control wild-type and mutant eggs were incubated in 1X E3 pronase-free buffer. To finish dechorionation, embryos were washed with 1X E3 buffer three times by gentle trituration using a Pasteur pipette, and examined at gastrula stage under a stereomicroscope. Cleaving rate was calculated using the Prism 7 (GraphPad) software.

### Microscopy, imaging and image processing

Live specimen images were captured using a Leica MZ 12–5 and Zeiss Stemi 305 stereomicroscope equipped with a Leica IC80 HD and Zeiss Axiocam 208 HD camera, respectively, and control software on an Apple Macintosh Computer. Histological section images were obtained using a Zeiss AXIOSKOP microscope and ProgRes® Mac CapturePro 2.6 (Jenoptik) software. Fluorescent labeled z-stacks were obtained using a Zeiss LSM 780 or 880 confocal microscope and the Plan-Apochromat 10×/0.45, the Plan-Apochromat 20×/0.8, or the Plan-Apochromat 40×/1.2 immersion water objectives.

For transmission electron microscopy, whole wild-type and *krang*^*p30ahub*^ ovaries were dissected and fixed in 2.5% glutaraldehyde, 2.0% paraformaldehyde, and 0.1 M sodium cacodylate overnight at 4°C. Washes, post-fixation, dehydration, embedding, thin sections, and staining were performed by using standard methods at the UPenn EM core facility. Stained sections were examined with a JEOL JEM 1010 electron microscope.

Images were processed using Fiji and Adobe Photoshop CC 2017 software.

### Data values

All individual data points for graphs in all figures or of data presented are included in spreadsheets in **S1 Data Values.**

### Protein sequence analysis and molecular modeling of the zebrafish Ap5m1 and Krang proteins

The similarity of the amino acids in the sequence alignments were scored using the BLOSUM substitution matrix with the software Multiseq [122].

The protein sequences of zebrafish Ap5m1 (Accession XP_005158951.1) and Krang/Kiaa0513 (Accession AAH74034) were searched in public non-redundant databases using iterated PSI-BLAST v.2.2.32+ (3^rd^ iteration), entries with ‘hypothetical’, ‘putative’, ‘modeled’ and ‘environmental samples’ were filtered out using entrez tools [123]. The resulting 270 sequences were filtered with a cut-off minimum of 20% identity and E-score of 1E-5, including full and partial alignments. Most of these sequences consisted of uncharacterized proteins for both Ap5m1 and Krang, respectively. The distance matrix was calculated using MUSCLE v.3.8 [123]. Neighbor joining protein sequence-based phylogenetic tree of Krang/Kiaa0513 homologs was handled using FigTree v1.4.2 (http://tree.bio.ed.ac.uk/software/figtree/). The tree was reduced from its original size by allowing a maximal sequence difference (Max Seq Difference) in fraction of mismatched residues of 0.85, resulting in 179 sequences. Bootstrap values from 500 iterations are shown for each node in the resulting cladogram (S4 Fig).

The Ap5m1 and Krang proteins secondary structure, solvent accessibility, flexibility, among other properties, were determined online using PredictProtein Server [124]. The tertiary structure of Ap5m1 and Krang were modeled online using I-TASSER Protein Structure and Function Prediction Server v.4.3 [125]. The Ap5m1 N- and C-terminal domains were defined based on the tridimensional structure of the best model. For Ap5m1 C-terminal domain modeling and structure alignment, the top ranked model based on the crystal structure of AP4m1 [49]; Protein Data Bank accession no. 3l81.PDB) was selected. The overall Root Mean Square Deviation (RMSD) of the protein structure alignment between zAp5m1 and hAp4m1 C-terminal domains was 2.45 Å, with an alignment length of 227 amino acids used in the superposition. The structural alignment was made using the TM-align server [126]. To generate 3D representations of the Ap5m1 (zebrafish), AP4m1 (human) and Krang (zebrafish) proteins, we utilized Visual Molecular Dynamics (VMD) 1.9.3 software.

## Supporting information

S1 Fig Lysis, CG behavior and embryo development in the opaque egg mutants. **A.** Eggs from wild-type, *blac*^*p25bdth*^ and *blac*^*p26ahubg*^ females at 40 mpf. **B.** Hematoxylin and Eosin stained sections of intact dissected ovaries (top row) and stage III oocytes (bottom row). Wild type (n = 12 ovaries), *p33bjta* (n = 11 ovaries), *ovy*^*p35aluc*^ (n = 10 ovaries) and *blac*^*p25bdth*^ (n = 14 ovaries) were phenotypically comparable and all oogenesis stages can be found. Overall, stage III mutant oocytes appeared comparable in size and general morphology to wild-type oocytes. However, under higher magnification discernible defects in YG size were evident compared to wild-type counterparts. Scale bar = 1.1 mm (A), 95 μm (B), 210 μm (D).
(TIF)

S2 Fig *Ap5m1* gene sequencing and intron retention in the *ovy*^*p37ad*^ mutant transcript. **A.** *ap5m1* cDNA PCR amplification schematic. The relative positions of the different primers used are above the *ap5m1* gene. Three different color-coded pairs of primers are shown. Exons are represented as numbered red boxes. **B.** Sequenced exon1-intron1 and intron1-exon2 junctions of the mutant *ap5m1* cDNA showing the donor splice site mutation, G to A, in intron 1 (black asterisk). **C.** Insertion of intron 1–2 found in the *ovy*^*p37ad*^ mutant allele. Predicted STOP codon encoded by the *ovy*^*p37ad*^ mutant *ap5m1* transcript suggests that the mutant transcript would produce a structurally incomplete protein lacking the C-terminal portion of the AP5m1 protein.
(TIF)

S3 Fig CGs and embryonic phenotype in *krang* mutants, pronase treatment, chorion structure analysis, and the mutation consequence. **A.** Confocal z-projections (60 μm depth) of acid fixed and MPA stained wild-type and mutant activated eggs. MPA staining reveals intact CGs of wild-type and mutant eggs at 20 mpa. CGs persist in *krang* eggs, revealing that the release of their content is compromised. In addition, numerous small CGs were retained after activation in eggs from these mutant females. **B.** Scatter plots of CG mean and median size in the DLR of wild-type and *krang* unactivated and activated eggs. Note different y-axis scales of the plots. Data are means ± SEM. *p = 0.0079 in a nonparametric statistical Mann-Whitney test. mpa, minutes post activation. **C.** Top: Graphs showing cleavage (left) and survival (right) of cleaving wild-type and mutant embryos. Two wild-type and 9 *krang* females were analyzed. Bottom: Wild-type control blastula were all normal at 1 dpf (n = 64/64, left panel). Most mutant embryos failed to develop beyond blastula stage (n = 64/82). Mutant blastulae gave rise to 1-dpf embryos (right panel) with a variable phenotype: wild-type-like (n = 10/82), and reduced body axis (8/82). **D.** Bars graph showing the cleavage percentage of pronase untreated and treated *krang* embryos. Fertilized wild-type and mutant eggs were subjected to pronase incubation (Treated; n = 60 (wild-type embryos) and n = 245 (mutant embryos), 2 wild-type and 4 *krang* females). Control cleaving percentage was obtained by incubating wild-type and *krang* eggs in pronase-free medium (Untreated; n = 60 (wild-type embryos) and n = 281 (mutant embryos), 2 wild-type and 4 *krang* females). **E.** Confocal micrographs of DAPI-stained fertilized zygotes showing normal chromosome segregation during the first mitosis (wild type (n = 13) and mutant (n = 14)). **F.** Ultrastructural analysis of the chorion morphology in wild-type (n = 12) and *krang* (n = 14) oocytes. Boxes show magnified areas revealing different electron density aspects of the chorionic zones I, II and III. **G.** *krang* mutation results in a frameshift in the coding sequence, which generates a premature stop codon (in red) following 24 aberrant amino acids and a truncated protein. Scale bar = 40 μm (A), 8.6 μm (E, left column), 2 μm (E, right column).
(TIF)

S4 Fig Evolutionary fate of Krang across the animal kingdom. Cladogram generated by analyzing Krang amino acid sequence showing its phylogenetic conservation among metazoans. Color-coded organisms are representative members of invertebrate and vertebrate phyla/classes. The numbers at the bases of the branches indicate bootstrap values obtained from 500 iterations. The scale bar on the bottom represents distances in residue substitutions per site.
(TIF)

S5 Fig CRISPR/Cas9-based mutation of zebrafish *krang* perturbs egg activation and embryo development. **A.** Schematic of the zebrafish *krang*/*kiaa0513* locus and CRISPR/Cas9 targeted region (red rectangle). Exons are shown as green boxes and introns as black lines. Sizes are not to scale. The 14 nt deletion and premature STOP codon generated by the mutation are shown. **B.** Representative images of mutant early embryos laid by transheterozygous females. Normal and defective chorion elevation in wild-type and mutant embryos, respectively, are indicated (black arrows). Notice that mutant early embryos display altered blastoderm formation. Scale bar = 311 μm.
(TIF)

S6 Fig Characterization of the 1-day post-fertilization and cell morphology *kazu* phenotype. **A.** *kazu*^*p26thbd*^ early embryo exhibits a smaller blastodisc phenotype and severely defective cytoplasmic segregation. Most *kazu*^*p26thbd*^ mutant embryos die at about 5 hpf and survivors may give rise to embryos with a reduced body. **B.** DAPI, α-tubulin, and centrosome staining showing abnormal blastoderm formation. Cell boundaries and mitotic figures are not observed in most of the mutant (n = 25/33) compared to wild-type (n = 30/35) embryos examined. Notice the formation of a syncytial nuclei layer in the 5 hpf *kazu*^*p26thbd*^ embryo. hpf, hours post-fertilization. Scale bar = 750 μm (A), 180 μm (B, left column), 150 μm (B, right column).
(TIF)

S1 Video 3D representation of cortical granules from both wild-type and *krang*^*p30ahub*^ mutant eggs at 20 mpa. The Z-stack images of the cortical granules were rendered in 3D using the 3D script plugin in Fiji. Scale bar = 10μm.
(AVI)

S1 Table Primers for genotyping mutants and for *ap5m1* gene mutation sequencing. (DOCX)

S1 Data Values All data points for graphs in all figures or of data presented are listed in spreadsheets. (XLSX)
