# Supplementary figures and images for: Maternal regulation of the vertebrate oocyte-to-embryo transition
Source: PLoS Genet. 2024 Jul 25;20(7):e1011343. doi: 10.1371/journal.pgen.1011343 (PMC11302925; doi:10.1371/journal.pgen.1011343)

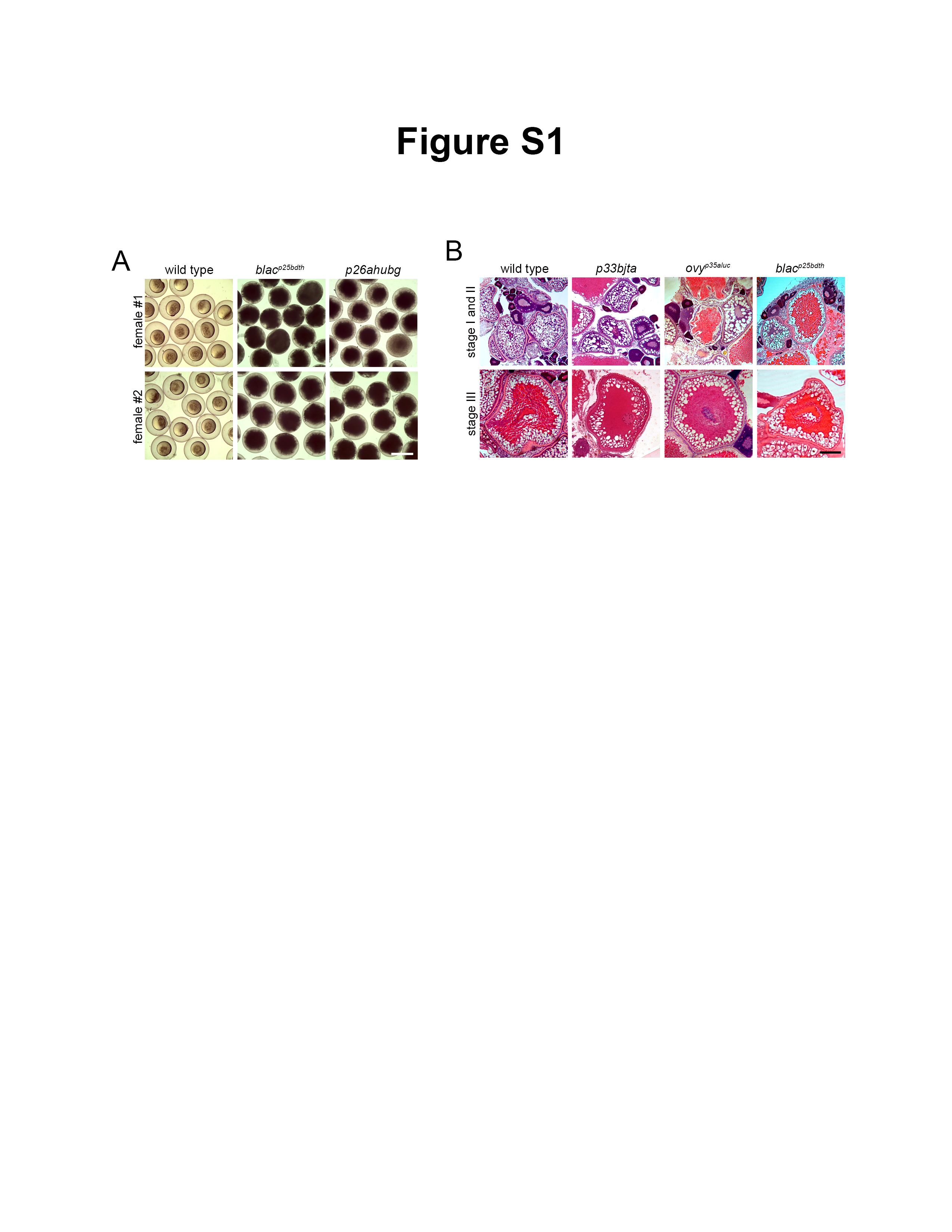

Supplement: S1 Fig — A. Eggs from wild-type, blacp25bdth and blacp26ahubg females at 40 mpf. B. Hematoxylin and Eosin stained sections of intact dissected ovaries (top row) and stage III oocytes (bottom row). Wild type (n = 12 ovaries), p33bjta (n = 11 ovaries), ovyp35aluc (n = 10 ovaries) and blacp25bdth (n = 14 ovaries) were phenotypically comparable and all oogenesis stages can be found. Overall, stage III mutant oocytes appeared comparable in size and general morphology to wild-type oocytes. However, under higher magnification discernible defects in YG size were evident compared to wild-type counterparts. Scale bar = 1.1 mm (A), 95 μm (B), 210 μm (D). (TIF) [file pgen.1011343.s001.tif]

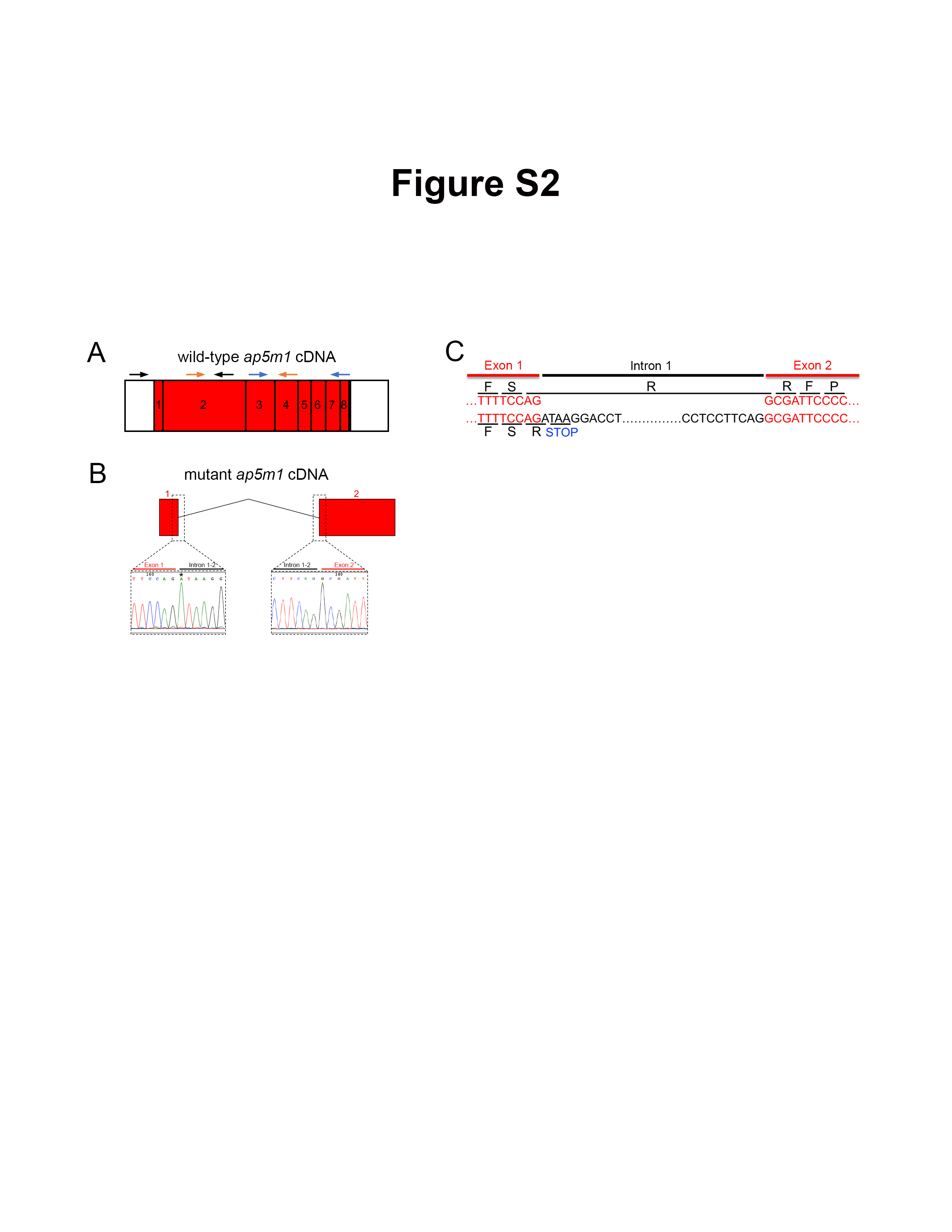

Supplement: S2 Fig — A. ap5m1 cDNA PCR amplification schematic. The relative positions of the different primers used are above the ap5m1 gene. Three different color-coded pairs of primers are shown. Exons are represented as numbered red boxes. B. Sequenced exon1-intron1 and intron1-exon2 junctions of the mutant ap5m1 cDNA showing the donor splice site mutation, G to A, in intron 1 (black asterisk). C. Insertion of intron 1–2 found in the ovyp37ad mutant allele. Predicted STOP codon encoded by the ovyp37ad mutant ap5m1 transcript suggests that the mutant transcript would produce a structurally incomplete protein lacking the C-terminal portion of the AP5m1 protein. (TIF) [file pgen.1011343.s002.tif]

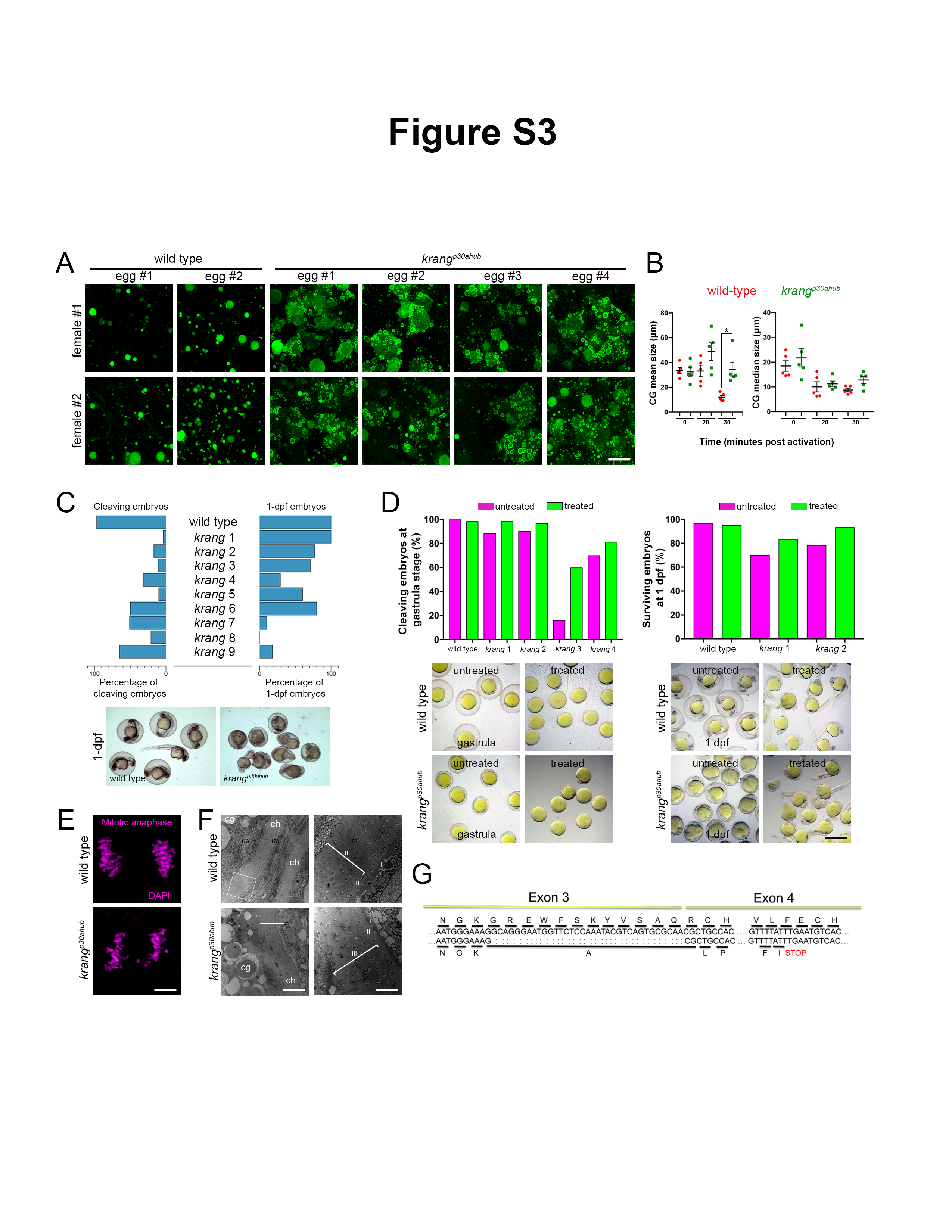

Supplement: S3 Fig — A. Confocal z-projections (60 μm depth) of acid fixed and MPA stained wild-type and mutant activated eggs. MPA staining reveals intact CGs of wild-type and mutant eggs at 20 mpa. CGs persist in krang eggs, revealing that the release of their content is compromised. In addition, numerous small CGs were retained after activation in eggs from these mutant females. B. Scatter plots of CG mean and median size in the DLR of wild-type and krang unactivated and activated eggs. Note different y-axis scales of the plots. Data are means ± SEM. *p = 0.0079 in a nonparametric statistical Mann-Whitney test. mpa, minutes post activation. C. Top: Graphs showing cleavage (left) and survival (right) of cleaving wild-type and mutant embryos. Two wild-type and 9 krang females were analyzed. Bottom: Wild-type control blastula were all normal at 1 dpf (n = 64/64, left panel). Most mutant embryos failed to develop beyond blastula stage (n = 64/82). Mutant blastulae gave rise to 1-dpf embryos (right panel) with a variable phenotype: wild-type-like (n = 10/82), and reduced body axis (8/82). D. Bars graph showing the cleavage percentage of pronase untreated and treated krang embryos. Fertilized wild-type and mutant eggs were subjected to pronase incubation (Treated; n = 60 (wild-type embryos) and n = 245 (mutant embryos), 2 wild-type and 4 krang females). Control cleaving percentage was obtained by incubating wild-type and krang eggs in pronase-free medium (Untreated; n = 60 (wild-type embryos) and n = 281 (mutant embryos), 2 wild-type and 4 krang females). E. Confocal micrographs of DAPI-stained fertilized zygotes showing normal chromosome segregation during the first mitosis (wild type (n = 13) and mutant (n = 14)). F. Ultrastructural analysis of the chorion morphology in wild-type (n = 12) and krang (n = 14) oocytes. Boxes show magnified areas revealing different electron density aspects of the chorionic zones I, II and III. G. krang mutation results in a frameshift in the coding sequence [file pgen.1011343.s003.tif]

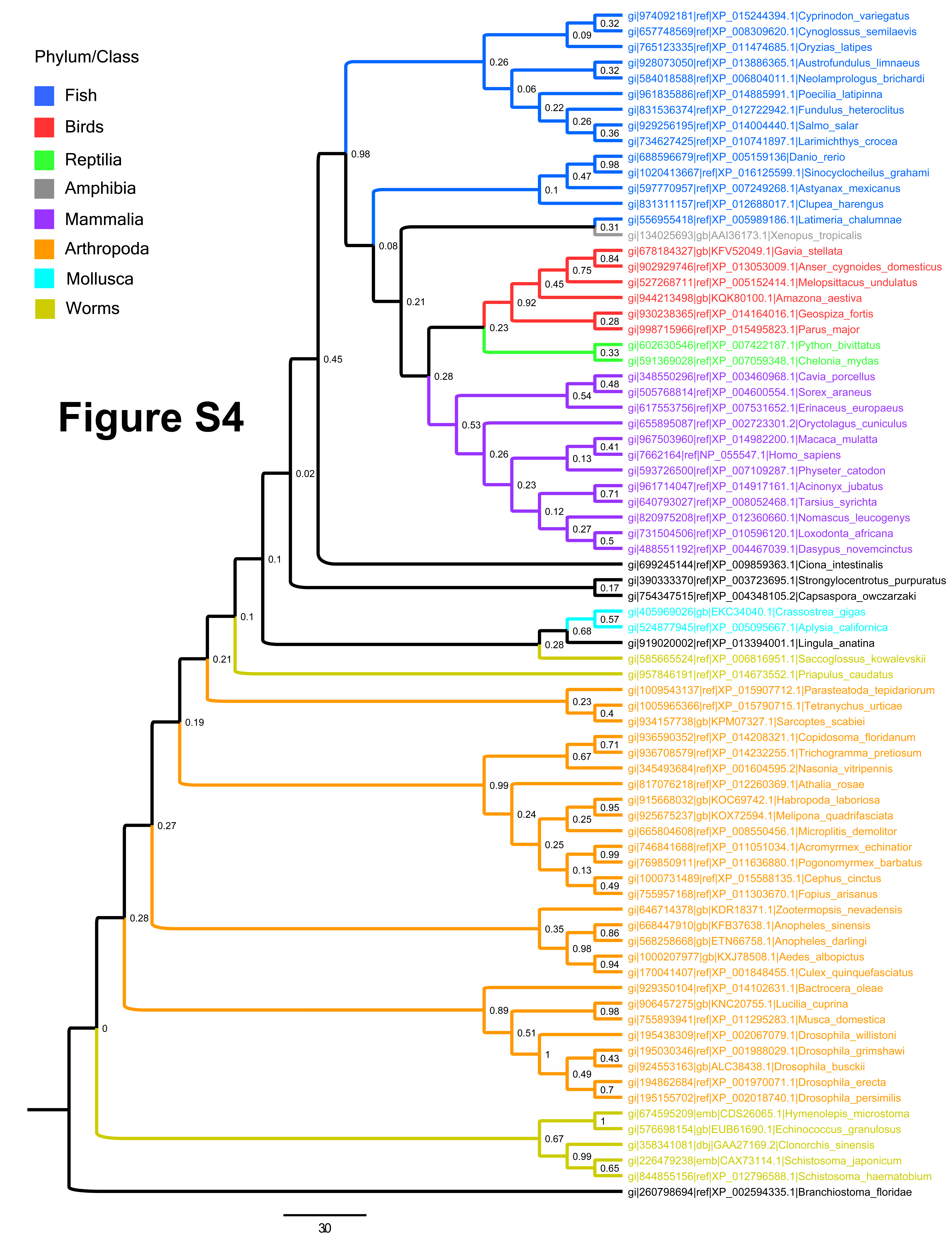

Supplement: S4 Fig — Cladogram generated by analyzing Krang amino acid sequence showing its phylogenetic conservation among metazoans. Color-coded organisms are representative members of invertebrate and vertebrate phyla/classes. The numbers at the bases of the branches indicate bootstrap values obtained from 500 iterations. The scale bar on the bottom represents distances in residue substitutions per site. (TIF) [file pgen.1011343.s004.tif]

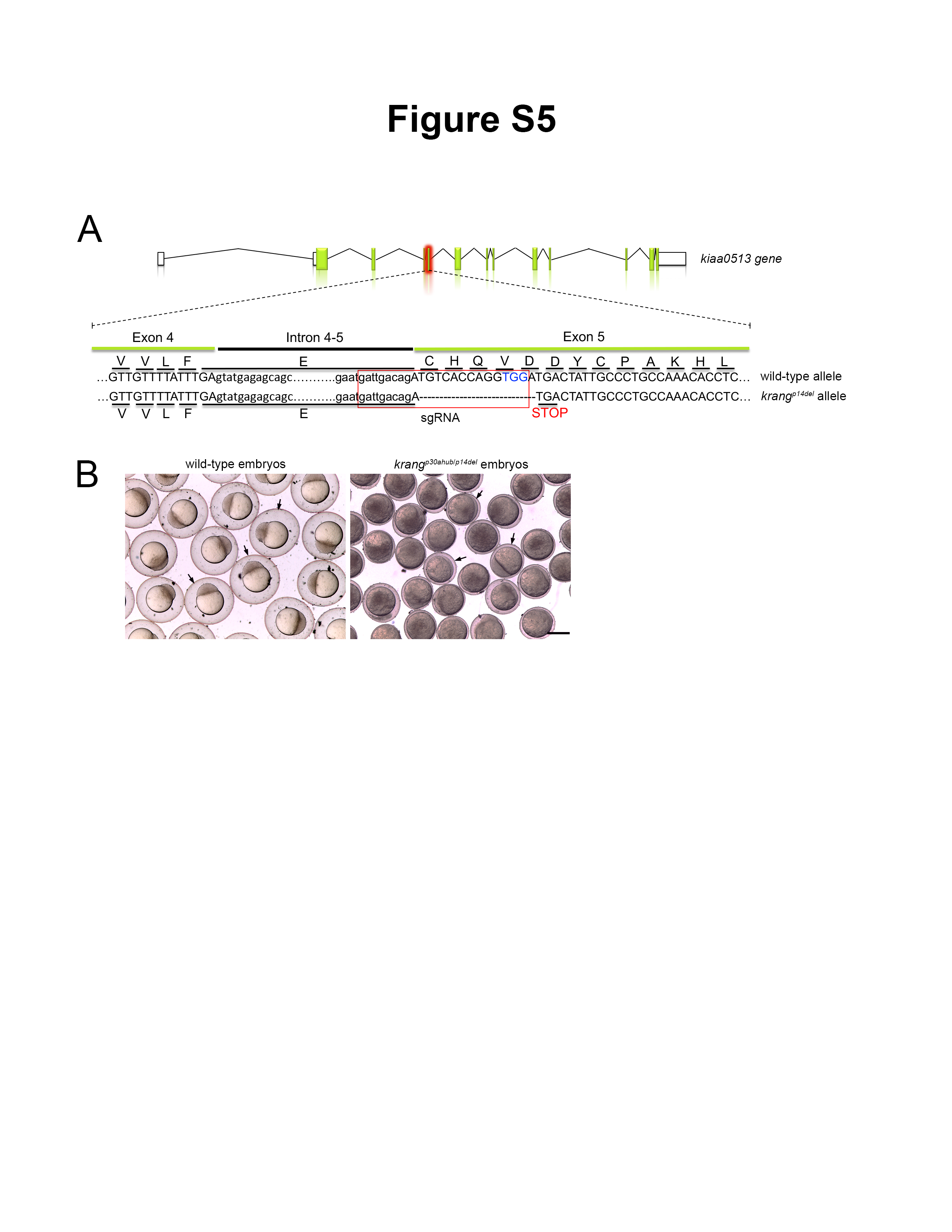

Supplement: S5 Fig — A. Schematic of the zebrafish krang/kiaa0513 locus and CRISPR/Cas9 targeted region (red rectangle). Exons are shown as green boxes and introns as black lines. Sizes are not to scale. The 14 nt deletion and premature STOP codon generated by the mutation are shown. B. Representative images of mutant early embryos laid by transheterozygous females. Normal and defective chorion elevation in wild-type and mutant embryos, respectively, are indicated (black arrows). Notice that mutant early embryos display altered blastoderm formation. Scale bar = 311 μm. (TIF) [file pgen.1011343.s005.tif]

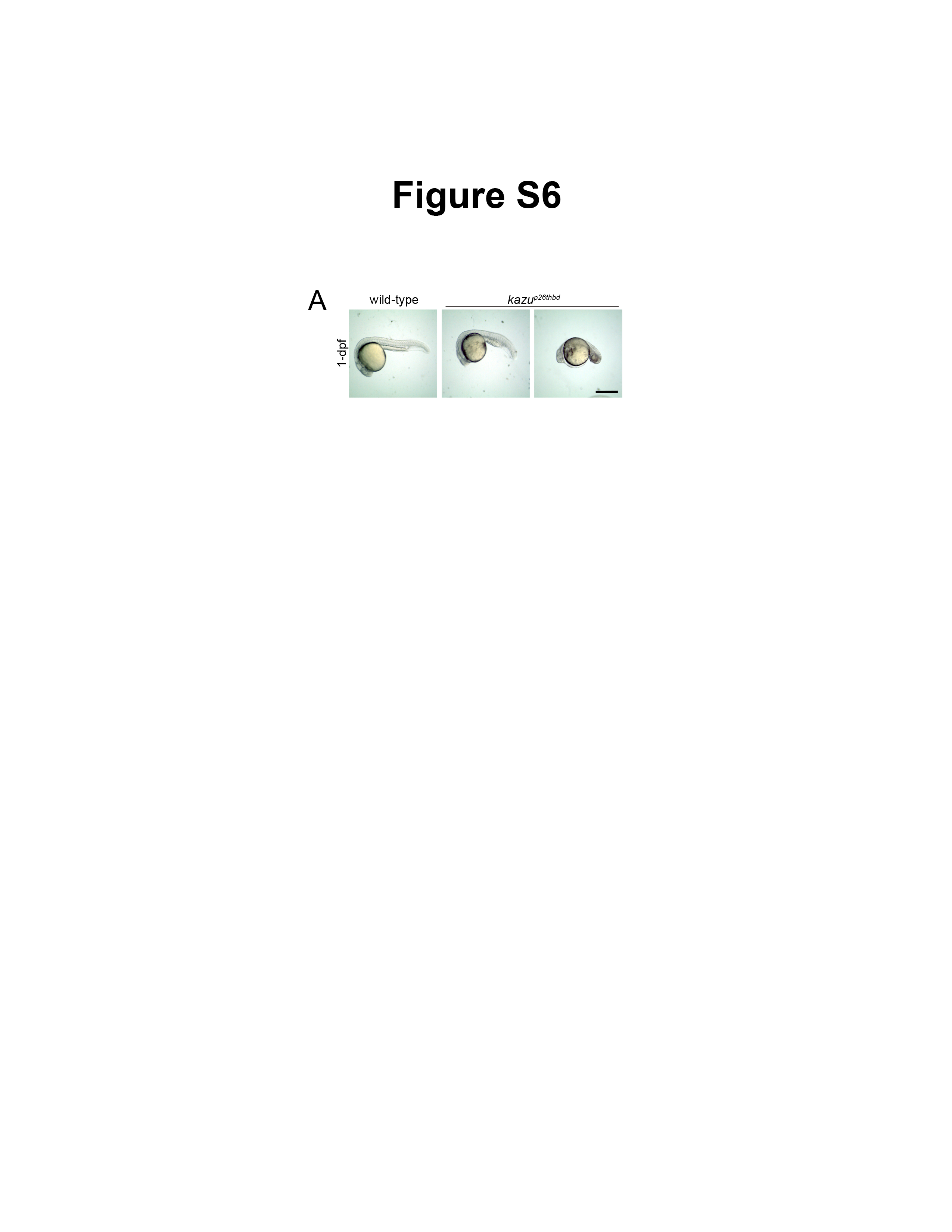

Supplement: S6 Fig — A. kazup26thbd early embryo exhibits a smaller blastodisc phenotype and severely defective cytoplasmic segregation. Most kazup26thbd mutant embryos die at about 5 hpf and survivors may give rise to embryos with a reduced body. B. DAPI, α-tubulin, and centrosome staining showing abnormal blastoderm formation. Cell boundaries and mitotic figures are not observed in most of the mutant (n = 25/33) compared to wild-type (n = 30/35) embryos examined. Notice the formation of a syncytial nuclei layer in the 5 hpf kazup26thbd embryo. hpf, hours post-fertilization. Scale bar = 750 μm (A), 180 μm (B, left column), 150 μm (B, right column). (TIF) [file pgen.1011343.s006.tif]
