# Supplementary material for: Maternal regulation of the vertebrate oocyte-to-embryo transition
Source: PLoS Genet. 2024 Jul 25;20(7):e1011343. doi: 10.1371/journal.pgen.1011343 (PMC11302925; doi:10.1371/journal.pgen.1011343)
Supplement: S1 Table — (DOCX) [file pgen.1011343.s008.docx]

**Table S1. Primers for genotyping mutants and for *ap5m1* gene mutation sequencing.**

| **Primer** | **Sequence** |
| --- | --- |
| z7170 | Forward Primer: 5’-GGCGAATAGGATTCGACAAA-3’  Reverse Primer: 5’-TCATGGAGATGAGTGAGTTGCT-3’ |
| z8980 | Forward Primer: 5’-GCCCCAGGGTAACATTTAAC-3’  Reverse Primer: 5’-GTTGGGTGCTGGTTTTGACT-3’ |
| z8703 | Forward Primer: GGCTGAGGATCATGTTTCGT  Reverse Primer: GGGTGAATGTATGACATTTTGG |
| *ap5m1*.amplicon1 | Forward Primer: 5’-CGGTCAGTCAGTGTTGTTATCG-3’  Reverse Primer: 5’-CTACAGCAGAAGGCACCAAT-3’ |
| *ap5m1*.amplicon2 | Forward Primer: 5’-AATCCTAGCCTGCCTTCCTCTTG-3’  Reverse Primer: 5’- GGTAGGAGCCAAGAATTGGT-3’ |
| *ap5m1*.amplicon3 | Forward Primer: 5’-CATTCTTGTCCATCCCTGTGTG-3’  Reverse Primer: 5’-GTATCCAGAAGAGACAGGAGC-3’ |
| *ap5m1*.5’end | Forward Primer: 5’-CGGTCAGTCAGTGTTGTTATCG-3’  Reverse Primer: 5’-GTCTGCTTCTTTCAGGTGAGTG-3’ |
| *ap5m1*.3’end | Forward Primer: 5’-TGAATGGCTCTCTGAAGAGG-3’  Reverse Primer: 5’-CTACAGCAGAAGGCACCAAT-3’ |
| *ovy^p35aluc^* | 5’-CTCTACATCATCAAAATATTTTTTCTTTGCCTCCTTCAG  GCGATTCCCCA[C/A]AGTTGAGTTCCGTGCTAAATCCTTG  GCTGGGTCTCATTATGTGGCCGTTC-3’ |
| *ovy^p37caed^* | 5’-TGGGTTGTCTCACACGAAAAGGGTGAATCTGGAAAAG  TACGGTTTTCCAG[G/A]TAAGGACCTTGTATATAATTGAC  TATTAGCATAGCTGATAGCTTTTGCTA-3’ |
| CR925798-1 | Forward Primer: 5’-TTTTTCATTTACTGGTATTGAAAGTG-3’  Reverse Primer: 5’-TGTACAGAAATTGGGGGAAAA-3’ |
| *krang^p30ahub^* | 5’-GAGGAGAAGGCTCGCTTTGGAGAGCTCTGTACTGGA  GACAATGGGAAAGG[C/T]AGGGAATGGTTCTCCAAATAC  GTCAGTGCGCAACGCTGCCAC-3’ |
| HRM | Forward Primer: 5’-TGGTGCAGTCTTTCGCTGTT-3’  Reverse Primer: 5’-AGGTGTTTGGCAGGGCAATA-3’ |
| *krang*_Exon4-Intron5-6 | Forward Primer: 5’-CGCTGCCACTCCAAATG-3’  Reverse Primer: 5’-GACACAATAATGGGCAGTACC-3’ |
